# Spectral mapping of brain functional connectivity from diffusion imaging

**DOI:** 10.1038/s41598-017-18769-x

**Published:** 2018-01-23

**Authors:** Cassiano O. Becker, Sérgio Pequito, George J. Pappas, Michael B. Miller, Scott T. Grafton, Danielle S. Bassett, Victor M. Preciado

**Affiliations:** 1 Department of Electrical and Systems Engineering, University of Pennsylvania, Philadelphia, USA; 2 Department of Industrial and Systems Engineering, Rensselaer Polytechnic Institute, Troy, USA; 3 Department of Psychological and Brain Sciences, University of California at Santa Barbara, Santa Barbara, USA; 4 Department of Bioengineering, University of Pennsylvania, Philadelphia, USA

## Abstract

Understanding the relationship between the dynamics of neural processes and the anatomical substrate of the brain is a central question in neuroscience. On the one hand, modern neuroimaging technologies, such as diffusion tensor imaging, can be used to construct structural graphs representing the architecture of white matter streamlines linking cortical and subcortical structures. On the other hand, temporal patterns of neural activity can be used to construct functional graphs representing temporal correlations between brain regions. Although some studies provide evidence that whole-brain functional connectivity is shaped by the underlying anatomy, the observed relationship between function and structure is weak, and the rules by which anatomy constrains brain dynamics remain elusive. In this article, we introduce a methodology to map the functional connectivity of a subject at rest from his or her structural graph. Using our methodology, we are able to systematically account for the role of structural walks in the formation of functional correlations. Furthermore, in our empirical evaluations, we observe that the eigenmodes of the mapped functional connectivity are associated with activity patterns associated with different cognitive systems.

## Introduction

Understanding the relationship between the dynamics of neural processes and the anatomical substrate of the brain is a central question in neuroscientific research^1^. Modern neuroimaging technologies, such as diffusion imaging^2,3^, allow researchers to track white matter streamlines linking cortical and subcortical structures. This information can be conveniently represented in terms of a *structural graph* representing direct anatomical connections^4,5^, between distinct brain regions. Complementary information can be acquired with functional neuroimaging techniques such as functional magnetic resonance imaging (fMRI)^6,7^, which measures time-dependent neural activity in the form of blood-oxygenation-level-dependent (BOLD) signals^8^. Temporal correlations between BOLD signals (averaged over representative brain parcels) can then be used to build a *functional connectivity* matrix, which unveils patterns of global coordination among various brain regions^9^. Prior studies offer preliminary evidence that whole-brain functional connectivity is shaped by the structural graph of anatomical connections^10,11^, yet the extent of this relationship in the human brain is not well understood.

An interesting problem in this context is understanding how functional connectivity emerges from the structural brain graph. This is a challenging problem for several reasons. First, the activity of brain regions that are not directly connected by structural links can be strongly correlated due to indirect structural walks along which signals propagate^12^. Second, the propagation of these signals is influenced, in a nontrivial manner, by the length and number of white matter streamlines in these walks^9^. Furthermore, it is unclear how signals propagating over different structural walks interfere or interact with each other to induce a global pattern of temporal correlations. Several studies have attempted to overcome these difficulties by mapping the functional connectivity of the human brain from the structural graph of anatomical connections. These approaches can be classified in the following major groups: (*i*) those performing a direct statistical comparison between the structural graph and the functional connectivity^9,10,13,14^, (*ii*) those based on numerical simulations of brain activity and connectivity^15–18^, (*iii*) operator-based propagator formulations of neural dynamics^19,20^, (*iv*) approximation of a nonlinear mapping between structural and functional connectivity using Taylor’s approximation theory^21^; and (*v*) those using graph-theoretical properties of the structural graph as mappings of functional connectivity^12,22^. While these studies have made important progress, it remains challenging to accurately map an individual’s functional connectivity from their structural brain graph.

In this article, we introduce a methodology based on spectral graph theory^23^ to map functional connectivity in the resting state (i.e., when a subject is at rest) from the structural graph using a versatile nonlinear mapping. Furthermore, using our methodology, we are able to systematically account for the role of indirect structural walks in the generation of functional correlations.

In what follows, we describe this methodology and illustrate its performance on neuroimaging data from the Human Connectome Project (HCP)^24^, using state-of-the-art acquisition and pre-processing methodologies. In our evaluations, we use structural graphs and functional matrices obtained from 44 different subjects measured non-invasively while at rest. In both cases, each node represents an anatomically defined parcel or brain region defined according to the Glasser^25^ multimodal parcellation atlas, which includes 360 cortical regions of interest. Using diffusion weighted imaging (DWI) and probabilistic tractography, we build the edges of the structural graph using the normalized number of streamlines connecting brain regions. The topology of the structural graph can be conveniently represented as an adjacency matrix^26^, denoted by *S*, where rows and columns are indexed by brain parcels, with entries being a function of the total number of streamlines between each pair of brain parcels. On the other hand, the functional connectivity matrix is computed using functional magnetic resonance imaging (fMRI) of blood-oxygenation-level-dependent (BOLD) time signals^8^. For each brain region, we extract a representative time series using the frequency-filtered average BOLD signal^9^ across the region. An entry [*F*]_*ij*_ in the functional connectivity matrix *F* is the Pearson’s correlation coefficient between signals extracted from regions *i* and *j*.

**Figure 1:**
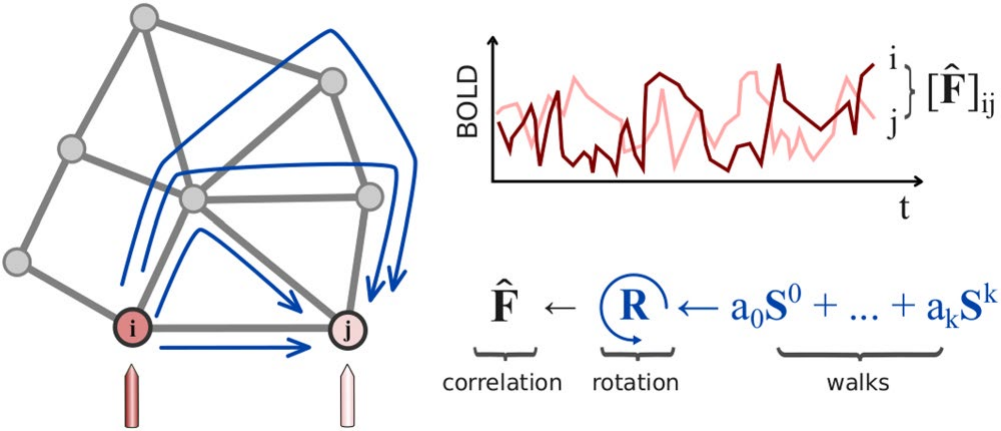
Approach schematic. The entry (*i*, *j*)-th entry of the functional connectivity matrix *F*, denoted by [*F*]_*ij*_, represents the correlation between the BOLD time signal components measured from two brain parcels corresponding to nodes *i* and *i* in the functional graph. The (*i*, *j*)-th entry of the *l*-th power of the structural matrix *S*, denoted by ${[{S}^{l}]}_{ij}$, accounts for walks of length *l* connecting nodes *i* and *j* in the structural graph. In the figure above, several walks of different lengths connecting nodes *i* and *j* are indicated by blue arrows. We propose to reconstruct the functional connectivity matrix *F* using a weighted sum of powers of the structural matrix, denoted by ${a}_{0}{S}^{0}+\ldots +{a}_{k}{S}^{k}$, as well as a change of coordinates (described by the rotation matrix *R*) aiming to align the eigenmodes of the mapped functional matrix $\hat{F}$ with those of *F*.

Using tools from spectral graph theory^23^, we propose a technique to map the functional connectivity matrix *F* of a subject at rest from his/her structural adjacency matrix *S*. Our mapping is comprised of two stages. In the first stage, we compute a weighted combination of the powers of the structural adjacency matrix *S* (see Fig. 1). As we discuss in the Materials and Methods section, the *l*-th power of *S* accounts for structural walks of length *l* connecting different brain regions. In practice, we truncate this weighted sum of powers at a particular value *k*, which represents the maximum length of the structural walks taken into account in the functional mapping. In the second stage of our mapping process, we perform a change of coordinates aiming to align the eigenmodes of the structural matrix *S*, which describe the spatial distribution of white matter streamlines, with the eigenmodes of the functional connectivity matrix *F*, whose values capture patterns of functional activity in different regions of the brain. Therefore, each functional eigenvector can be described as a weighted combination of the structural eigenvectors (see Materials and Methods section for further details). As a result, we obtain a functional mapping $\hat{F}$ whose entries are a nonlinear combination of measurements related to structural walks of lengths up to *k*, and overall distribution of the white matter streamlines crisscrossing the brain.

**Figure 2:**
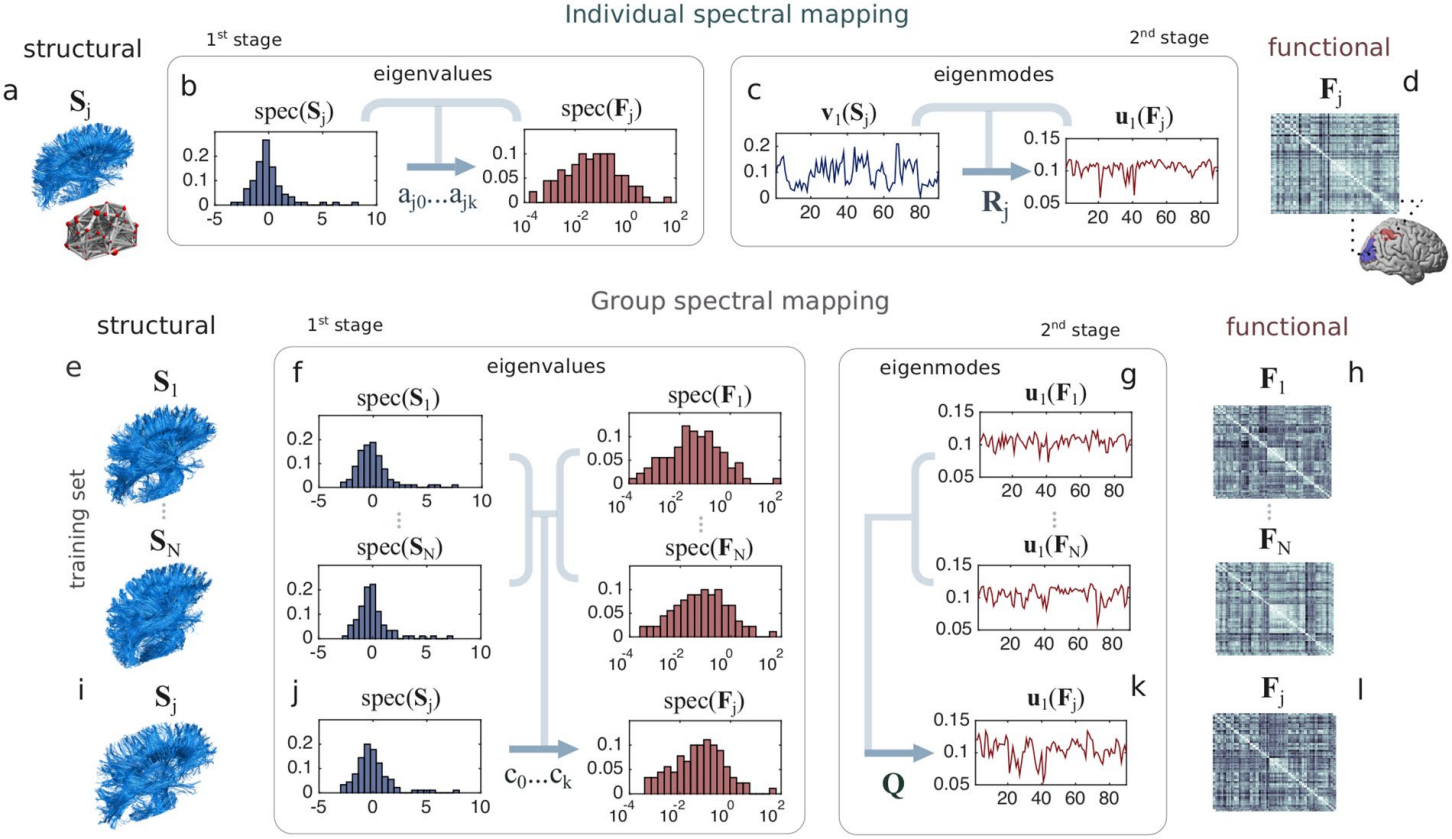
Spectral mapping method. In the *individual spectral mapping* problem (**a**–**d**), we map the functional connectivity matrix *F*_*j*_ of an individual *j* at rest (**d**) from his/her structural connectivity (**a**). Our mapping is based on a two-stage process. In the first stage, we use a polynomial transformation of order *k* (characterized by the coefficients ${a}_{j0},\ldots ,{a}_{jk}$) to map the eigenvalues of *F*_*j*_ from those of *S*_*j*_. In (**b**), we include the histogram of the eigenvalues of *S*_*j*_ and *F*_*j*_ for the *j*-th individual (in linear and log-linear scale, respectively). In the second stage, we use a rotation matrix *R*_*j*_, specific to individual *j*, to infer the eigenmodes of *F*_*j*_ from those of *S*_*j*_ (as illustrated in (**c**), for the first eigenmode). In the lower part of the figure (**e**–**l**), we illustrate the *group spectral mapping* problem, in which we find a ‘universal’ mapping, valid for a whole group of individuals. For that purpose, we specify a training set composed by structural connectivity graphs (**e**) and their corresponding functional connectivity matrices (**h**). In the first stage (**f**), we find a common polynomial transformation (characterized by ${c}_{0},\ldots ,{c}_{k}$), using the eigenvalues of structural and functional connectivity matrices in the training set. In the second stage (**g**), we find a common set of eigenmodes described by a matrix *Q* for all the individuals in the training set. Finally, using both the polynomial transformation (**j**) and the rotation (**k**), we estimate the functional connectivity matrix *F*_*j*_ of a subject *j* (**l**) from his/her structural graph (**i**).

We compute the parameters of this two-stage functional mapping by solving an optimization problem aiming to maximize the quality of the functional mapping $\hat{F}$. In our experimental evaluations, we measure the functional mapping quality using the Pearson correlation between the entries of the mapped functional matrix $\hat{F}$ and those of the actual functional matrix *F* (see the Materials and Methods section). Hereafter, we refer to this optimization problem as the *spectral mapping problem*. In this paper, we study two different types of spectral mapping problems (represented in Fig. 2). First, we consider the problem of finding a ‘personalized’ functional mapping for each subject. We refer to this problem as the *individual spectral mapping problem*. Second, we consider the problem of finding *common functional eigenmodes* across the different subjects. Simply speaking, in the individual spectral mapping problem we aimed to determine structural to functional dependencies specific to a subject, whereas now we seek common resting state functional patterns among the subjects. We refer to this problem as the *group spectral mapping problem*. In what follows, we solve both spectral problems and illustrate the performance of our approach on two different neuroimaging datasets.

## Materials and Methods

### Subjects

A total of 44 individuals were randomly selected from the HCP 1200 release^24^. According to the coordinating institution, subject recruitment procedures and informed consent forms, including consent to share de-identified data, were approved by the Washington University institutional review board. The participants gave written consent, and the relevant procedures were carried out in accordance with the institutional review board approval and procedures, in accordance with relevant guidelines and regulations.

### Connectivity matrices

The connectivity matrices considered in our experiments are built using neuroimaging data from the HCP dataset^24^, whose acquisition protocols and pre-processing pipelines have been fully described^27^ and extensively discussed in dedicated literature^28^. The structural matrices were constructed by applying probabilistic tracking^29^ with crossing fibers using the probtrackx tool from FSL^30^, using the pre-processed estimated diffusion parameters included in the HCP 1200 release, which were generated by FSL’s bedpostx. The number of streamlines between regions was used as a metric of structural connectivity strength. Each region in the structural graph corresponds to a localized brain area in the Glasser-360 parcellation^25^. The matrices were symmetrized by averaging entries resulting from tracking in reciprocal directions, and were normalized by dividing all entries by the overall maximum weight. Functional MRI data considered resting state sessions, whose runs of approximately 15 minutes comprised 1200 samples acquired with a 3 T Gradient-echo EPI and TR = 720 ms. Cardiac signals were measured by a pulse oximeter, sampled at 400 Hz and synchronized with the scanner. Respiratory signals were measured by an elastic respiratory belt transducer, also sampled at 400 Hz, i.e. 288 samples per frame of functional image. As a post-processing step on the fMRI time series, both cardiac and respiratory down-sampled signals were regressed out of time series data through linear regression. Functional connectivity matrices were built as follows. For each brain region in the Glasser-360, we extracted a representative time-series using the bandpass (0.06–0.125 Hz) filtered time components of the average BOLD signal in each region. The (*i*, *j*)-th entry of the functional connectivity matrix is given by the Pearson’s correlation coefficients between the representative time-series of regions *i* and *j*. We adopt a methodology that considers two partitions of the dataset, which we refer to as *in-sample* and *out-of-sample*. Briefly, in the individual spectral mapping problem, the in-sample and out-of-sample functional connectivity matrices are obtained by randomly sub-sampling the samples of the time response before calculating the in-sample and out-of-sample correlation matrices. In contrast, for the group spectral mapping problem, the in-sample and out-of-sample matrices are obtained by correspondingly partitioning the set of subjects, while considering the whole set of time samples for the functional matrix of each subject. Both definitions are explained in detail in subsections ‘Individual spectral mapping’ and ‘Group spectral mapping’, respectively.

### Spectral graph theory

Consider a structural brain graph with *n* nodes and weighted edges. We denote by *S* the *n *× *n* adjacency matrix of the structural graph, i.e., the entry [*S*]_*ij*_ is the weight of the edge connecting nodes *i* and *j*. We define a walk of length *l* from node *i*_0_ to *i*_*l*_ in the graph as an ordered sequence of *l* + 1 nodes, $({i}_{0},{i}_{1},\ldots ,{i}_{l})$, such that the pair of nodes $\{{i}_{r-1},{i}_{r}\}$ are connected for all $r=\mathrm{1,}\ldots ,l$. We denote by ${{\mathcal{P}}}_{{i}_{0},{i}_{l}}^{l}$ the set of all walks of length *l* from *i*_0_ to *i*_*l*_ in the structural graph. Given a walk $p=({i}_{0},{i}_{1},\ldots ,{i}_{l})$, we define the weight of the walk as the following product:

1 $$\omega (p)=[S{]}_{{i}_{0}{i}_{1}}{[S]}_{{i}_{1}{i}_{2}}\ldots {[S]}_{{i}_{l-1}{i}_{l}}\mathrm{.}$$

A fundamental result in spectral graph theory relates the *l*-th power of the adjacency matrix *S* with walks of length *l* in the structural graph, as follows:

2 $${[{S}^{l}]}_{ij}=\sum _{p\in {{\mathcal{P}}}_{i,j}^{l}}\omega (p)\mathrm{.}$$

In other words, the (*i*, *j*)-th entry of *S*^*l*^ is equal to the sum of the weights of all walks of length *l* from *i* to *j*. In our solution to the spectral mapping problem, we use a weighted sum of powers of *S* up to order *k*; in particular, we use the matrix ${\rm{\Omega }}=f(S)={a}_{0}{S}^{0}+\ldots +{a}_{k}{S}^{k}$. According to (2), the (*i*, *j*)-th entry of Ω is equal to a weighted sum over all walks from *i* to *j* of length up to *k*. Furthermore, our functional mapping $\hat{F}$ is a similarity transformation of Ω; in particular, $\hat{F}=R{\rm{\Omega }}{R}^{\top}$ with *R* being a rotation matrix, whose effect is described next.

### Rotation matrix

In the context of functional and structural connectivity matrices, the rotation matrix *R* can be understood as follows. Recall that the entries of the connectivity matrix *F* correspond to the correlation coefficients of the time-series associated with the BOLD signal associated with each possible pair of brain regions. As such, the functional eigenmodes are aligned with patterns of neural activity, as discussed in subsection ‘Common functional eigenmodes are revealed by group spectral mapping’. Correspondingly, the structural eigenmodes capture spatial patterns of propagation throughout streamlines connecting different brain regions^31^. Now, let the eigenvectors of *S* (respectively, *F*), denoted by ${\{{v}_{i}\}}_{i\mathrm{=1}}^{n}$ (respectively., ${\{{u}_{i}\}}_{i\mathrm{=1}}^{n}$) form the columns of the matrices $V=[{v}_{1}|{v}_{2}|\ldots |{v}_{n}]$ (respectively, $U=[{u}_{1}|{u}_{2}|\ldots |{u}_{n}]$). We now define the rotation matrix *R* such that $R=U{V}^{\top}$ (see detailed mathematical derivation in Subsection 3.1 the Supplementary Information), which establishes a mapping between the structural eigenmodes and the functional eigenmodes. Using the rotation matrix, we then build a weight matrix $W={V}^{\top}RV$ such that $U=VW$. Therefore, the columns (eigenmodes) of *U* and *V* are related via the following linear combination:

3 $${u}_{i}=V{w}_{i}={w}_{1i}{v}_{1}+{w}_{2i}{v}_{2}+\ldots +{w}_{ni}{v}_{n},$$

where *w*_*i*_ is the *i*-th column of *W* and *w*_*ji*_ is the *j*-th component of this column vector. In summary, from the rotation matrix *R* we can directly build a matrix *W* that allows us to map each functional eigenmode (*u*_*i*_) as a linear combination of structural eigenmodes (*v*_*j*_).

### Individual spectral mapping

In this work, we propose a technique to map the functional connectivity matrix *F* of a subject at rest from his/her structural adjacency matrix *S*. As mentioned above, we propose a mapping parameterized as follows:

4 $$\hat{F}=R\,f(S){R}^{\top},$$

where $f(S)={\sum }_{i\mathrm{=0}}^{k}{a}_{i}\,{S}^{i}$ and *R* is an orthogonal rotation matrix. To find the values of the parameters ${\{{a}_{i}\}}_{i\mathrm{=0}}^{k}$ and *R*, we propose to minimize the quadratic (or Frobenius) norm of the difference between *F* and $\hat{F}$, defined as:

5 $${\Vert \hat{F}-F\Vert }_{ {\mathcal F} }^{2}=\sum _{i\mathrm{=1}}^{n}\sum _{j\mathrm{=1}}^{n}{({[\hat{F}]}_{ij}-{[F]}_{ij})}^{2}\mathrm{.}$$

In other words, given the *n* × *n* functional and structural matrices of an individual, *F* and *S*, as well as the value of the maximum order *k*, we solve the following optimization problem:

6 $$\mathop{\,{\rm{minimize}}\,\,}\limits_{{\{{a}_{i}\}}_{i\mathrm{=0}}^{k},R}{\Vert R(\sum _{i\mathrm{=0}}^{k}{a}_{i}\,{S}^{i}){R}^{\top}-F\Vert }_{ {\mathcal F} }^{2}\,{\rm{subject}}\,{\rm{to}}\,\,R{R}^{\top}={R}^{\top}R={I}_{n},$$

where the constraints guarantee *R* to be a rotation matrix. We show in the Supplementary Information that the solution to this optimization problem can be found as follows.

First, compute the eigenvalues and eigenvectors of *S* (respectively, *F*), denoted by ${\{{v}_{i}\}}_{i\mathrm{=1}}^{n}$ and ${\{{\lambda }_{i}\}}_{i\mathrm{=1}}^{n}$ (respectively, ${\{{u}_{i}\}}_{i\mathrm{=1}}^{n}$ and ${\{{\phi }_{i}\}}_{i\mathrm{=1}}^{n}$). Define the Vandermonde matrix

7 $$L=[\begin{array}{ccccc}1 & {\lambda }_{1} & {\lambda }_{1}^{2} & \cdots  & {\lambda }_{1}^{k}\\ 1 & {\lambda }_{2} & {\lambda }_{2}^{2} & \cdots  & {\lambda }_{2}^{k}\\ \vdots  & \vdots  & \vdots  & \ddots  & \vdots \\ 1 & {\lambda }_{n} & {\lambda }_{n}^{2} & \cdots  & {\lambda }_{n}^{k}\end{array}],$$

where the parameter *k* is the maximum order of the polynomial. Therefore, it can be shown (see Supplementary Information) that a solution pair $({\{{a}_{i}^{* }\}}_{i\mathrm{=0}}^{k},{R}^{* })$ to the optimization problem in (6) is given by ${({a}_{0}^{* },\ldots ,{a}_{k}^{* })}^{\top}=({L}^{\top}L{)}^{-1}{L}^{\top}\,\phi$ and ${R}^{* }=U{V}^{\top}$. Subsequently, the functional mapping is given by:

8 $$\hat{F}={R}^{* }(\sum _{i\mathrm{=0}}^{k}{a}_{i}^{* }{S}^{i}){({R}^{* })}^{\top}\mathrm{.}$$

Simply speaking, our mapping is the result of aligning both the eigenvalues and eigenvectors of a subject’s structural and functional connectivity matrices. Notice that, in general, such alignment would not be possible without considering the rotation matrix. Furthermore, since an *n* × *n* rotation matrix *R* has $n(n-\mathrm{1)/2}$ degrees of freedom^32^, the solution to (6) could in principle allow for overfitting due to the additional number of parameters in the mapping. To validate that this is not the case, we consider a version of the optimization problem (6) in which we constrain the rank of *R* to be equal to a given integer *m* (see Supplementary Information for more details). In this respect, we compute in subsection ‘Stability of spectral mapping’ the map for different values of *m*, whose results support our claim about the absence of overfitting in $\hat{F}$.

#### In-sample and out-of-sample dataset partitioning for the individual spectral mapping

In what follows, we explain how to generate in-sample and out-of-sample functional matrices to train our mapping and assess its quality. We start with a collection of bold signals ${b}_{r}=({b}_{r}(1),\ldots ,{b}_{r}(L){)}^{\top}$ of length *L *= 1200, where *b*_*r*_(*s*) denotes the *s*-th time sample of the average BOLD signal in the *r*-th brain region (where $r=\mathrm{1,}\ldots ,M$, according to the Glasser-360 atlas *M* = 360 regions). For each region *r*, we partition the signal vector *b*_*r*_ into two: one first vector ${b}_{r}^{(1)}$ including *L*/2 entries of *b*_*r*_ (chosen uniformly at random without repetition), and ${b}_{r}^{(2)}$, which includes the remaining entries of *b*_*r*_. Using the sets of vectors ${\{{b}_{r}^{(1)}\}}_{r\mathrm{=1}}^{M}$ and ${\{{b}_{r}^{(2)}\}}_{r\mathrm{=1}}^{M}$, we compute two functional correlation matrices; the *in-sample* matrix *F*^(1)^ and the *out-of-sample* matrix *F*^(2)^, where ${[{F}^{(1)}]}_{ij}$ (respectively, ${[{F}^{(2)}]}_{ij}$) is the Pearson correlation coefficient between ${b}_{i}^{(1)}$ and ${b}_{j}^{(1)}$ (respectively, ${b}_{i}^{(2)}$ and ${b}_{j}^{(2)}$). In our numerical experiments, we use *F*^(1)^ to find the optimal set of parameters for the mapping $\hat{F}$ (i.e., we use *F*^(1)^ to solve the optimization problem in (6)). In the main document, we use the out-of-sample matrix *F*^(2)^ to assess the quality of our mapping.

### Group spectral mapping

Consider a group of *N* individuals whose structural and functional matrices are given by the set of pairs ${\{({S}_{j},{F}_{j})\}}_{j\mathrm{=1}}^{N}$. In this mapping problem, our objective is to find a *common* mapping able to generate an approximation of *F*_*j*_ from *S*_*j*_, while addressing the following question: can a mapping be obtained in terms of *common functional eigenmodes* across the different subjects? Simply speaking, in the individual spectral mapping problem we aimed to determine structural to functional dependencies specific to a subject, whereas now we seek common resting state functional patterns among the subjects. Towards this goal, we propose to determine a mapping ${\hat{F}}_{j}=Q\,g({S}_{j})Q$, where $g({S}_{j})={\sum }_{r\mathrm{=0}}^{k}{c}_{r}{{\rm{\Lambda }}}_{j}^{r}$, with ${{\rm{\Lambda }}}_{j}={\rm{diag}}({\lambda }_{1}({S}_{j}),\ldots ,{\lambda }_{n}({S}_{j}))$ and *Q* is an orthogonal matrix encapsulating the common functional eigenvectors. In particular, notice that the structural eigenvectors of a specific subject are not considered, since we aim to find a common set of functional eigenmodes. Therefore, the parameters ${\{{c}_{r}\}}_{r\mathrm{=0}}^{k}$ and *Q* are the solution to the following optimization problem:

9 $$\mathop{\,{\rm{minimize}}\,\,}\limits_{{\{{c}_{r}\}}_{r\mathrm{=0}}^{k},Q}\sum _{j\mathrm{=1}}^{N}{\Vert Q(\sum _{r\mathrm{=0}}^{k}{c}_{r}\,{{\rm{\Lambda }}}^{r}){Q}^{\top}-{F}_{j}\Vert }_{ {\mathcal F} }^{2}\,{\rm{subject}}\,{\rm{to}}\,\,Q{Q}^{\top}={Q}^{\top}Q={I}_{n}\mathrm{.}$$

The numerical solution to this problem involves an iterative optimization procedure, which is described in Section 3.2 of the Supplementary Information.

#### In-sample and out-of-sample dataset partitioning for the group spectral mapping

Our dataset consists of a group of 44 subjects. In our experiment, we partition this group into two subgroups of equal size, while considering the full-length of the time series. We use the pairs of structural and functional matrices in the first subgroup (which we refer to as the *in-sample* set) to train the common mapping, and validate our results with the second subgroup (the *out-of-sample* set).

### Matrix correlation quality

In our empirical evaluations, we measure the similarity between two *n* × *n* square matrices *X* and *Y* using the *matrix correlation function*, denoted by ${\rm{ucorr}}(X,Y)$, and defined as the entry-wise correlation between the upper-triangular entries (excluding diagonal elements) of *X* and *Y*. In other words, if we build two vectors *x* and *y* of dimension $n(n-1)\mathrm{/2}$ by ‘vectorizing’ the upper triangular entries of *X* and *Y*, the matrix correlation function is simply the correlation between these two vectors.

### Computational implementation

We provide a publicly available computational implementation of the algorithms, allowing other researchers to evaluate and compare mapping results over other databases. The implementation is available for download at https://brainopt.github.io/spectral-mapping.

## Results

### Individual functional connectivity is mapped with high accuracy

**Figure 3:**
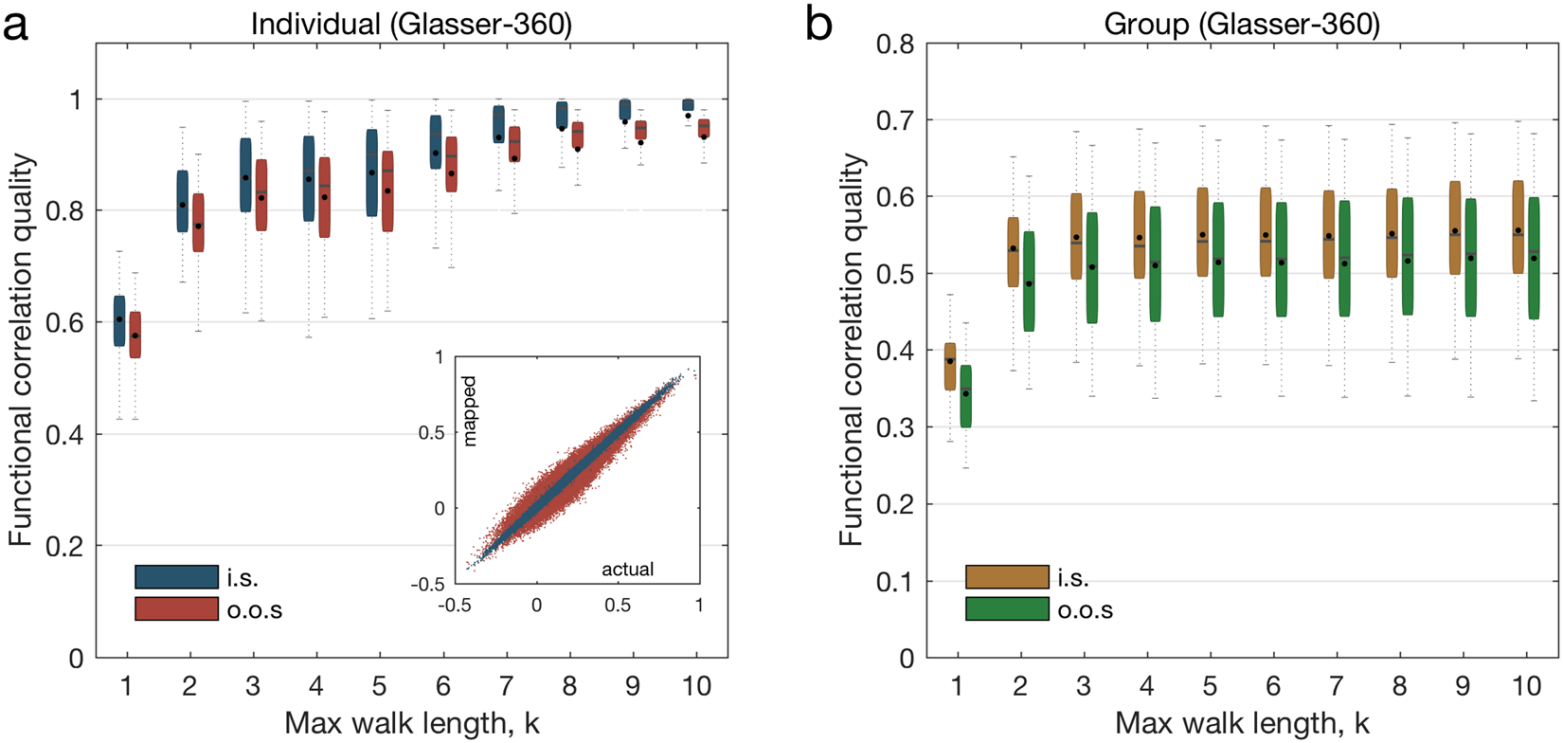
Spectral mapping performance (Glasser-360). We represent the evolution of the correlation quality between the mapped and the actual functional connectivity matrices when we vary the maximum length of the walks under consideration (denoted by the parameter *k*) for the individual and the group spectral mappings. In (**a**), we plot the evolution of the correlation quality evaluated over 3 different splits of the BOLD signal time-series samples in the training (in blue) and validation (in red) sets, for the individual spectral mapping. The inset in (**a**) includes a scatter plot of the entries of the mapped functional matrix $\hat{F}$ (values in the ordinates) versus the actual connectivity matrix *F* (values in the abscissae) when *k* = 8 for the individual with the median correlation quality. The ordinates of the blue (respectively, red) dots in the scatter plot correspond to the entries of the functional connectivity matrix used for training (respectively, validation). In (**b**), we plot the evolution of the correlation quality evaluated over 3 different splits of the BOLD signal time-series obtained from group spectral mapping in the training (in yellow) and validation (in green) sets.

We first focus our attention on the individual spectral mapping problem (pictorially represented in Fig. 2a–d, and mathematically described in the Materials and Methods section). In our numerical evaluations, we consider two different BOLD time series for each subject, which we construct as follows. Beginning with a single BOLD time series with 1200 time samples, we build one set of samples by randomly selecting half the samples from the original signal. The second set is built by choosing the other half of remaining samples. The first set is then used to generate an *in-sample* functional connectivity matrix to train the functional mapping. The second set is used to generate an *out-of-sample* functional connectivity matrix to validate the quality of the trained mapping. In a first set of experiments, we train and validate ‘personalized’ functional mappings for each one of the 44 subjects in the dataset. For each individual, we build a hierarchy of mappings with different values of *k*, where *k* ranges from 1 to 10. In other words, we gradually increase the maximum length of the structural walks being considered in the functional mapping. In Fig. 3a, we plot the distribution of the quality of the personalized functional mapping for both the training (in-sample) and the validation (out-of-sample) functional matrices. Using the in-sample data, the correlation level achieved by the functional mapping after training becomes consistently close to l as *k* increases above 8 (blue boxes in Fig. 3a). Using the out-of-sample data to validate the trained mapping, we observe that the quality of the mapping consistently increases with *k*, saturating at an average correlation of 0.9410 for *k* above 8.

**Figure 4:**
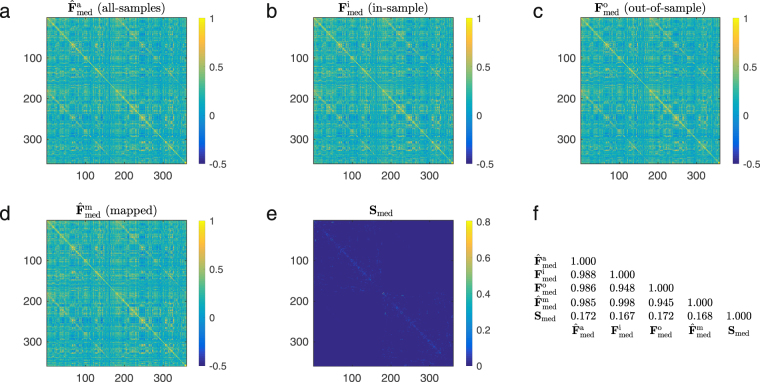
Structural and functional connectivity matrices for the Glasser-360 parcellation. We consider an individual whose mapped functional connectivity achieves the median correlation quality across all subjects, for parameter *k* = 8. In (**a**), we present the functional connectivity matrix obtained from all time samples. In (**b**) and (**c**), we show the empirical functional connectivity matrix for the same individual obtained from the in-sample and out-of-sample data, respectively. In (**d**) we display the mapped functional connectivity matrix using the individual spectral mapping, whereas in (**e**) we present its structural connectivity matrix. Lastly, (**f**) displays the pair-wise correlation between the above mentioned matrices, for all possible combinations.

Based on these results, we can quantify the role of structural walks of different lengths in the formation of the functional connectivity pattern. For example, since the median mapping quality for *k* = 1 is 0.5735 in the validation dataset, we conclude that direct structural walks of length l account for 0.5735 of the personalized mapping quality. Furthermore, the median mapping quality increases to 0.7747 when we also consider walks of length *k* = 2. Hence, we conclude that structural walks of length 2 account, in the median, for a 20.12% increment in the mapping quality. Similarly, as we gradually include structural walks of length 3 to 10 in the mapping, the median quality increases according to the following incremental percentages: 5.761%, 1.122%, 2.723%, 2.618%, 2.607%, 1.805%, 0.643%, and 0373%. Notice how the propagation of neural signals through short structural walks has the strongest influence in the resulting functional connectivity. In particular, for a value of *k* as low as 3, we note that median mapping quality is above 80% (0.8323). We also observe that the median mapping quality relatively saturates at $k\ge 8$. Therefore, walks of length 3 and up to 8 in the structural graph contain most of the information needed to map functional correlations, offering fundamental insight into the lengths of walks used for neural interactions. In the inset in Fig. 3a, we include a scatter plot to compare the entries of the actual functional connectivity matrix ${F}_{j}$ (values in the abscissae) with those of the mapped functional matrix ${\hat{F}}_{j}$ (values in the ordinates) when we choose *k* = 8 for the individual with the median correlation quality. The ordinates of the blue (respectively, red) dots correspond to the entries of the functional connectivity matrix used for training (respectively, validation). For illustration purposes, we also display in Fig. 4 structural and functional connectivity matrices of an individual whose mapped functional connectivity achieves the median correlation quality across all subjects.

**Figure 5:**
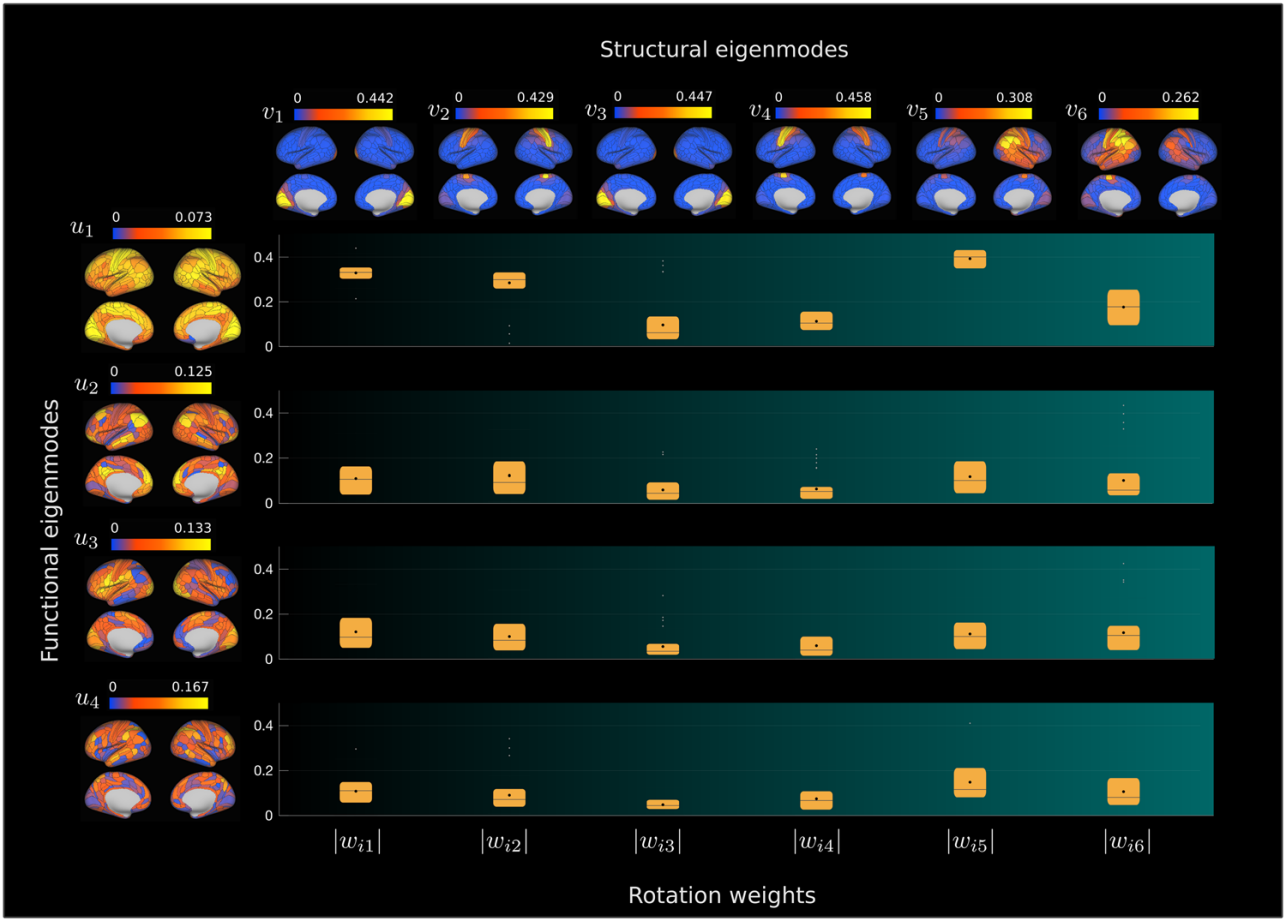
Decomposition of functional eigenmodes in terms of structural eigenmodes for the Glasser-360 parcellation. The absolute values of the components of functional eigenmodes *u*_*i*_ ($i=\mathrm{1,}\ldots \mathrm{,4}$) associated with the largest functional eigenvalues are vertically displayed on the left-hand side. In the top row, we display the average across individuals of the absolute values of the components of structural eigenmodes *v*_*i*_ ($j=\mathrm{1,}\ldots \mathrm{,6}$) associated with the largest structural eigenvalues. Additionally, we summarize the distribution across individuals of the first six absolute values of the weights *w*_*ij*_ (depicted as box plots) used to express each functional eigenmode *u*_*i*_ as a linear combination of structural eigenmodes *v*_*i*_, when all possible *n* = 360 structural modes in the parcellation are considered.

With regard to the rotation matrix, its role in relating the functional and structural eigenmodes can be visualized in terms of the projection of the eigenmodes on the cortical surface. Recall that the functional eigenmodes (describing the dynamic patterns of neural activity) can be represented as a linear combination of the structural eigenmodes (capturing spatial patterns of propagation). Such relationship is expressed in (3) and is illustrated in Fig. 5. The figure displays the actual distribution of weights obtained for the decomposition of the four functional eigenmodes associated with the largest functional eigenvalues in terms of the structural eigenmodes, along with their respective cortical projections.

### Group spectral mapping partially maps individual connectivity from common parameters

In addition to the individual spectral mapping problem, we also consider the *group spectral mapping problem*, in which we aim to find a ‘universal’ mapping able to estimate a representative functional connectivity matrix for a group of subjects. In other words, given the structural graphs and functional connectivity matrices of a group of individuals, we aim to find a *common* mapping to estimate the functional connectivity matrix for any individual in the group with the maximum possible overall correlation quality (see the Materials and Methods section for a technical description of this problem). From a neuroscientific perspective, this problem corresponds to understanding the role of common eigenmodes in neural interactions performed in many individuals. To evaluate the performance of the method, we partition the set of 44 subjects into two subsets: an in-sample subset of 22 individuals whose structural graphs and functional connectivity matrices are used to train the universal mapping, and an out-of-sample subset of 22 individuals used to evaluate the mapping quality. In this setup, we find the optimal parameters of the universal mapping for the in-sample training set when the maximum length of structural walks under consideration ranges from *k* = 1 to 10. In Fig. 3b, we plot the performance of the universal functional mapping for both the in-sample training set (yellow boxes) and the out-of-sample validation set (green boxes). Overall, the performance in the validation set is well aligned with the training set, and both tend to increase as we increase the value of *k*. As expected, the universal mapping presents a lower performance when compared with the personalized mappings, highlighting the existence of meaningful individual differences in how functional eigenmodes interact to compose functional dynamics. In particular, when the out-of-sample mapping case is considered, the best median performance stabilizes at around 0.5180 for the group case (while in the personalized case, it saturates at around 0.9410). It is interesting to conjecture that differences between the mapped and actual functional connectivity matrices may be related to the individual’s cognitive state in healthy subjects, or to individual differences in symptomatology in clinical populations.

### Spectral mapping exhibits robustness to parcellation and image acquisition techniques

**Figure 6:**
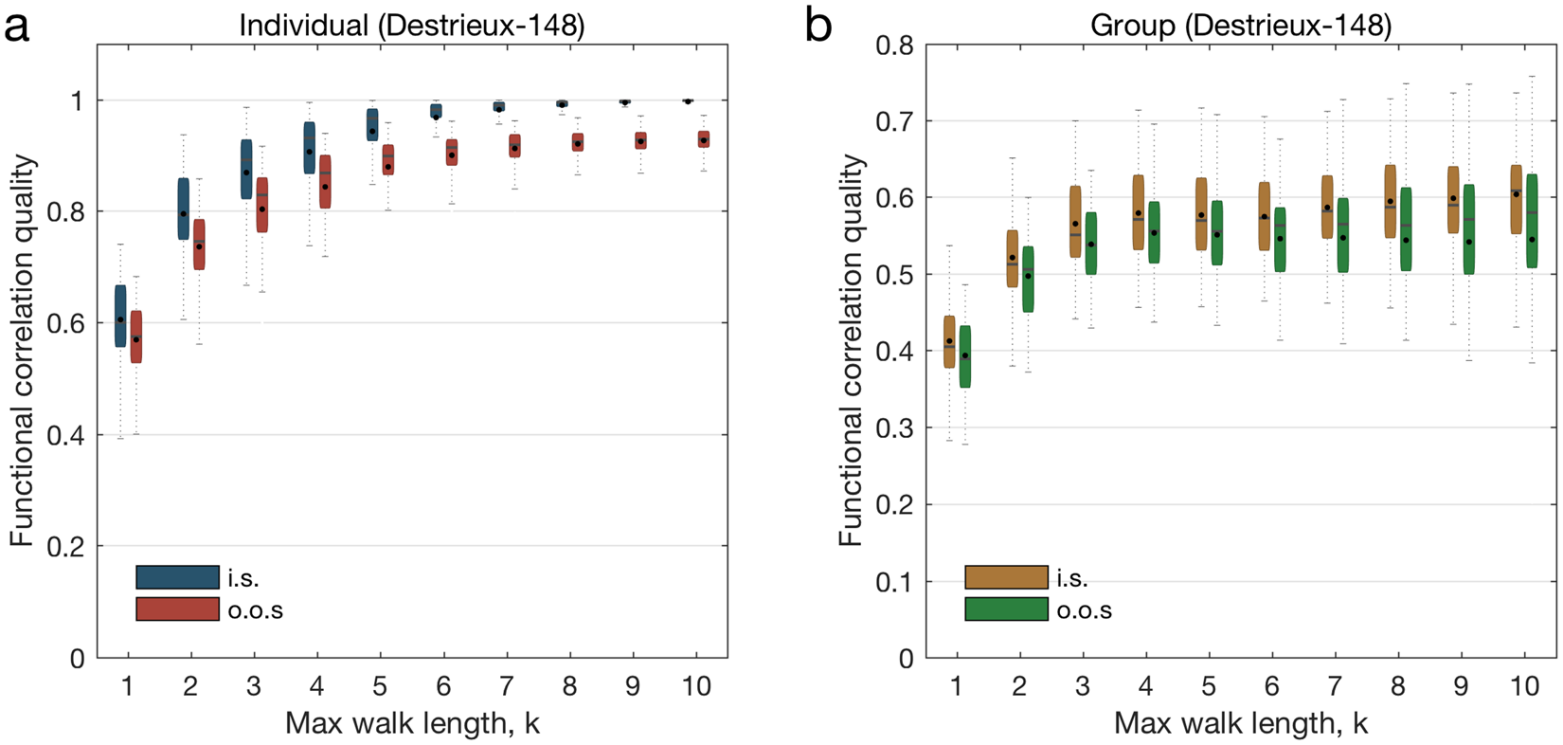
Spectral mapping performance (Destrieux-148). We represent the evolution of the correlation quality between the mapped and the actual functional connectivity matrices when we vary the maximum length of the walks under consideration (denoted by the parameter *k*) for the individual and the group spectral mappings, for the Destrieux-148^33^ parcellation. In (**a**), we plot the evolution of the correlation quality evaluated over 3 different splits of the BOLD signal time-series samples in the training (in blue) and validation (in red) sets, for the individual spectral mapping. In (**b**), we plot the evolution of the correlation quality evaluated over 3 different splits of the BOLD signal time-series in the training (in yellow) and validation (in green) sets, for the group spectral mapping.

**Figure 7:**
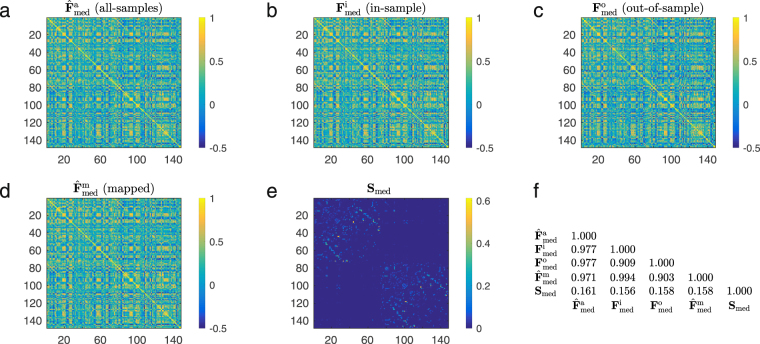
Structural and functional connectivity matrices for the Destrieux-148^33^ parcellation (148 regions). We consider an individual whose mapped functional connectivity achieves the median correlation quality across all subjects, for parameter *k* = 5. In (**a**), we present the functional connectivity matrix obtained from all time samples. In (**b**) and (**c**), we show the empirical functional connectivity matrix for the same individual obtained from the in-sample and out-of-sample data, respectively. In (**d**) we display the mapped functional connectivity matrix using the individual spectral mapping, whereas in (**e**) we present its structural connectivity matrix. Lastly, (**f**) displays the pair-wise correlation between the above mentioned matrices, for all possible combinations.

It is instructive to evaluate if the spectral mapping method achieves comparable performance with different parcellation and acquisition approaches to generating structural and functional connectivity matrices. To address this question, we performed the same analyses described in the previous section with an alternative anatomical atlas. More precisely, we consider connectivity matrices generated from a parcellation into 148 regions following morphological criteria^33^, which we refer to as Destrieux-148. The corresponding evolution of functional matrix correlation quality for the in-sample and out-out-sample sets, obtained for the individual and group spectral mappings, is presented in Fig. 6a,b. We note that it exhibits a similar behavior to the one obtained for the Glasser-360, with the correlation quality stabilizing at lower values of the parameter *k*, what can be mathematically explained by the comparatively smaller total number of regions in the parcellation. In particular, as with what was observed for Glasser-360, in the current case, the median mapping quality for *k* = 3 is above 80% (0.8290). Correspondingly, in Fig. 7 we display the structural and functional connectivity matrices of an individual whose mapped functional connectivity achieved the median correlation, considering all splits of the dataset. As a second set of experiments, we also considered an alternative dataset^9^, whose results are presented in the Supplementary Information. This dataset employs legacy single-tensor diffusion technology, which imposes significant limitations^34,35^, with respect to the tractography and estimation of structural connectivity matrices. These findings are therefore included exclusively to enable discretionary comparison with previous studies that use comparably limited datasets.

### Common functional eigenmodes are revealed by group spectral mapping

**Figure 8:**
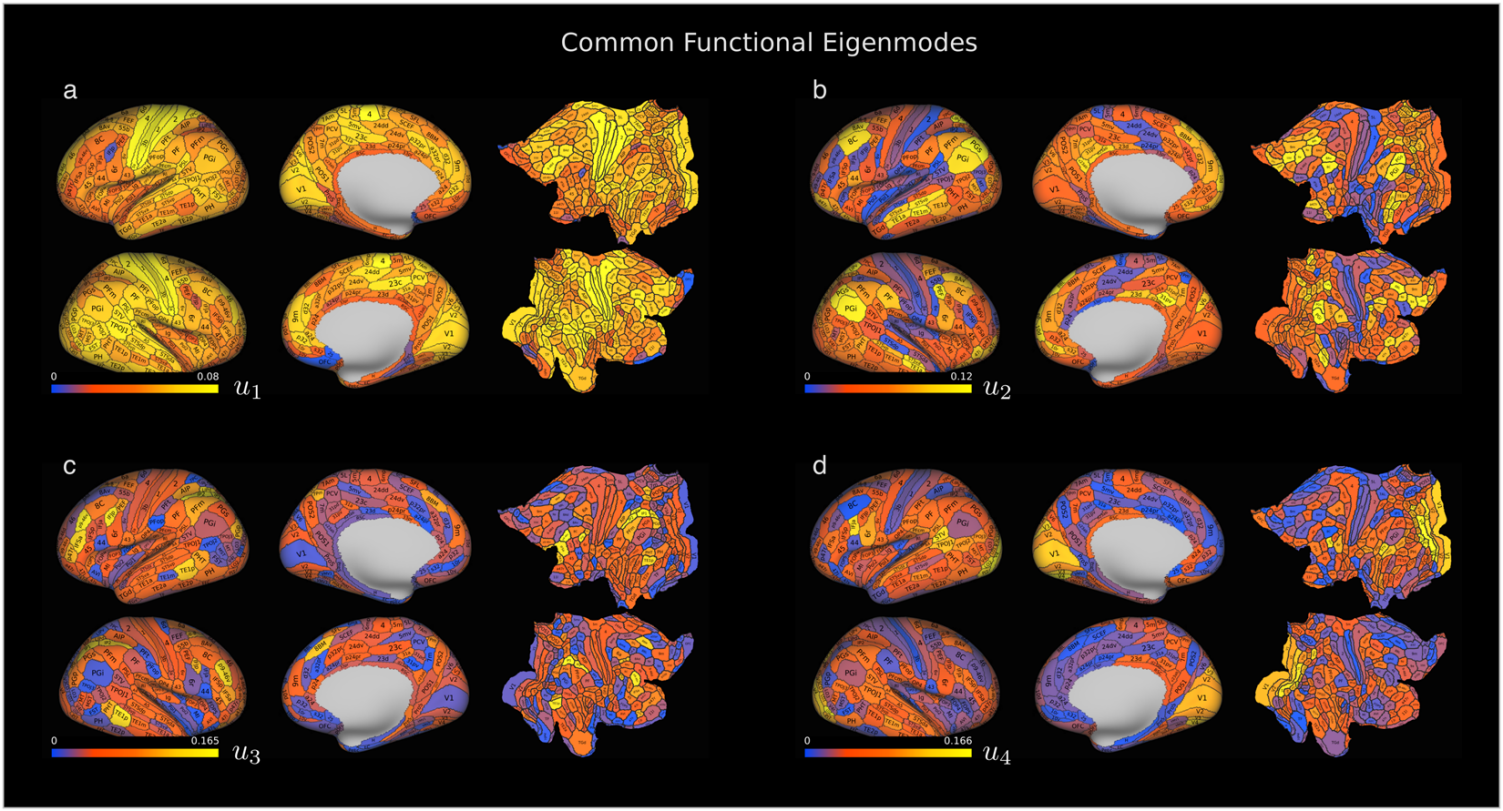
Eigenmodes of the mapped functional connectivity obtained from group spectral mapping. Lateral, medial and flat cortical surface renderings for the absolute values of the components of the eigenmodes associated with the four largest eigenvalues of the functional connectivity matrix generated by group spectral mapping. In (**a**), color plot for the first eigenmode (i.e., Bonacich centrality of the functional connectivity matrix). The blue areas (OFC, 25) are likely to be areas where signal is corrupted by missing data, distortion artifacts and other artifacts associated with imaging this area of the brain with fMRI. In (**b**), the color plot of the second eigenmode takes high values (yellow) over the posterior prefrontal cortex, parieto-occipito-temporal (POT) region and middle temporal gyrus. In (**c**), the third eigenmode takes high values over the primary and secondary visual cortices, left ventral premotor areas and inferior parietal cortex. In (**d**), the fourth eigenmode takes high values over most areas of the intraparietal sulcus and dorsolateral prefrontal cortex.

To investigate the neurophysiological drivers of these mappings, we depict in Fig. 8 brain surface activation maps representing the first four common eigenmodes of the mapped functional connectivity $\hat{F}$ (i.e., the eigenvectors of $\hat{F}$ associated with the four largest eigenvalues). In particular, the first eigenmode (Fig. 8a) represents the so-called Bonacich centrality of the functional connectivity matrix, which measures how ‘well-connected’ or ‘central’ a region is in the functional graph^36^. The displayed activation areas correspond to widespread cortical involvement. Dominant areas in the second eigenmode (Fig. 8b) include posterior prefrontal cortex, parieto-occipito-temporal (POT) region and middle temporal gyrus. These are all heteromodal cortex areas associated with higher cognitive processing. The third eigenmode (Fig. 8c) presents high values over primary and secondary visual cortices, left ventral premotor areas and inferior parietal cortex. These areas are all associated with vision and visually guided action planning. Finally, the fourth eigenmode (Fig. 8d) presents dominant contributions in most areas of the intraparietal sulcus and dorsolateral prefrontal cortex. These areas form an strongly connected prefrontal-parietal network involved in attention.

### Stability of spectral mapping

**Figure 9:**
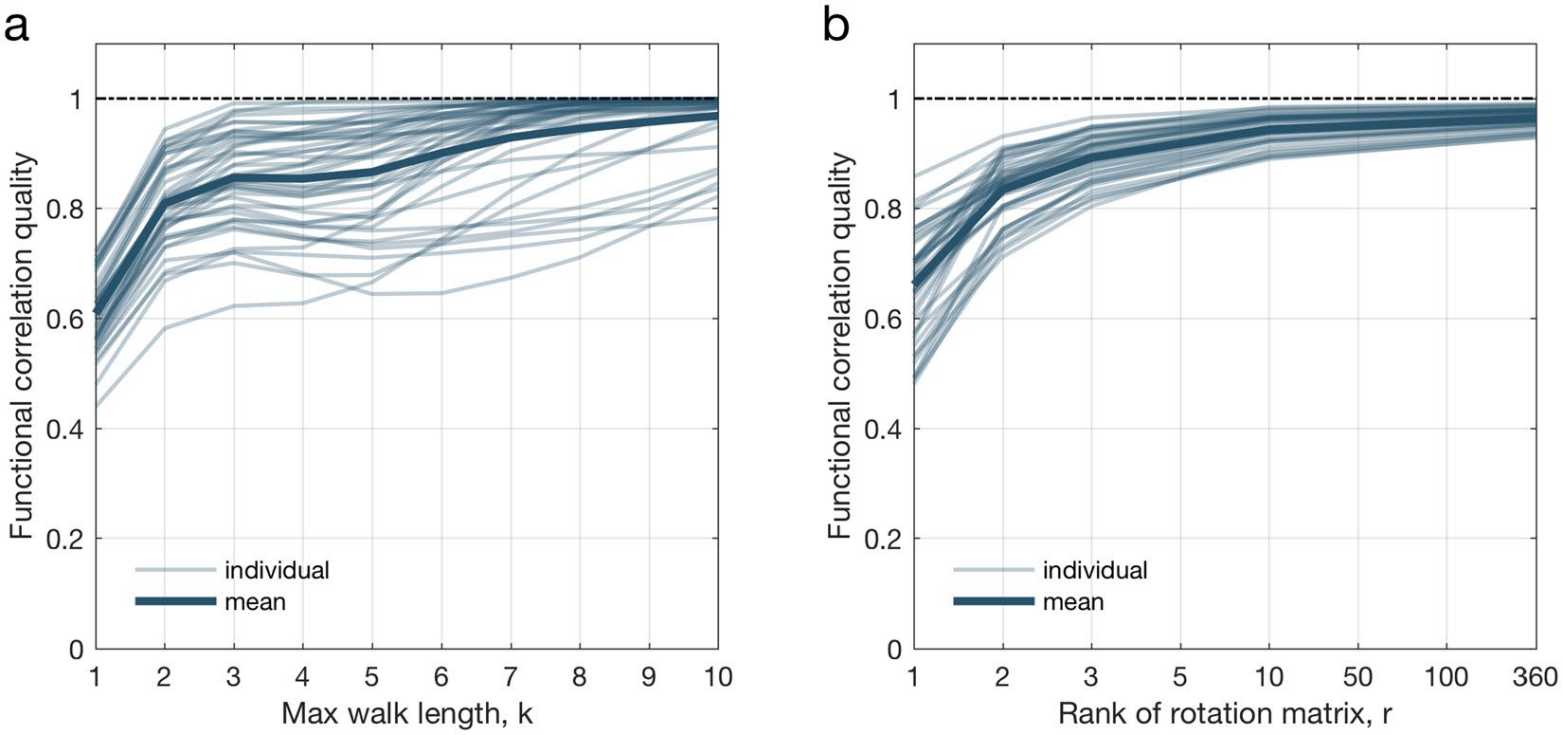
Stability of spectral mapping for individual spectral mapping (for all subjects). Thin lines denote the evolution of the functional correlation quality for each one of the 44 individuals in the dataset, whereas bold lines indicate the average over all thin lines. In (**a**) we plot the evolution of the quality for as we vary the value of the hyper-parameter *k*, while in (**b**) we plot the quality evolution as we vary the hyper-parameter *m*.

In what follows, we examine the stability of our mapping to ensure that the spectral mapping method is not overfitting the data. In this direction, we vary the number of degrees of freedom in the functional mapping using two hyper-parameters. The first hyper-parameter is the *order* of the maximum power of *S*, denoted by *k*, included in the first stage of our functional mapping (see Fig. 2). As previously mentioned, this value is equal to the maximum length of the structural walks considered by the functional mapping. The second hyper-parameter is the *rank m* of the rotation matrix *R* used in the second stage of the functional mapping (see Fig. 2). By reducing the rank of the rotation matrix, we restrict the number of eigenmodes of *S* that we aim to align with the eigenmodes of *F*. Computationally, this has the effect of reducing the number of free parameters associated with the rotation matrix *R*. In Fig. 9, we plot the influence of these hyper-parameters on the quality of the solution of the individual spectral mapping problem. This quality is measured as the correlation coefficient between the entries of the functional matrix *F* and the entries of the mapped functional matrix $\hat{F}$. Figure 9 contains subplots displaying the evolution of the functional correlation quality for all the 44 individuals in the dataset as we vary the hyper-parameters. In our evaluations, we observe that the functional mapping achieves a high correlation quality even for relatively low values of the hyper-parameters, indicating that it is possible to map the functional connectivity with a relatively low number of degrees of freedom.

### Perturbation analysis

Now, we propose to investigate the robustness of the individual spectral mapping with respect to noise in the structural matrices. First, for each structural and functional connectivity matrix pair (*S*,*F*) associated with a given individual, we obtain the set of parameters (a^*^, R^*^) obtained as a solution to (6). Then, we generate a perturbed matrix $\check{S}$ from the original structural matrix *S*, i.e., we adopt a random multiplicative model acting on each element of the structural matrix. This model has the desirable property of preserving the structural matrix sparsity, and producing perturbations which are, on average, proportional to the value of each element. Quantitatively, the perturbation model can be described as follows. For each element [*S*]_*ij*_ or the original structural matrix, we sample a perturbation value from a uniform distribution with support between (−*ρ*, *ρ*), i.e., ${[{\rm{\Delta }}]}_{ij}\sim {\rm{uniform}}(-\rho ,\,\rho )$, where *ρ* is a parameter that we vary. An entry of the perturbed matrix is, therefore, defined as

10 $${[\check{S}]}_{ij}=\,(1+{[{\rm{\Delta }}]}_{ij})[S{]}_{ij}.$$

Subsequently, we compute the (perturbed) mapped functional connectivity matrix $\check{F}$ using the set of parameters (a^*^, R^*^), i.e.,

11 $$\check{F}={R}^{\ast }(\sum _{i=0}^{k}{a}_{i}^{\ast }{\check{S}}^{i}){({R}^{\ast })}^{{\rm{\top }}}.$$

**Figure 10:**
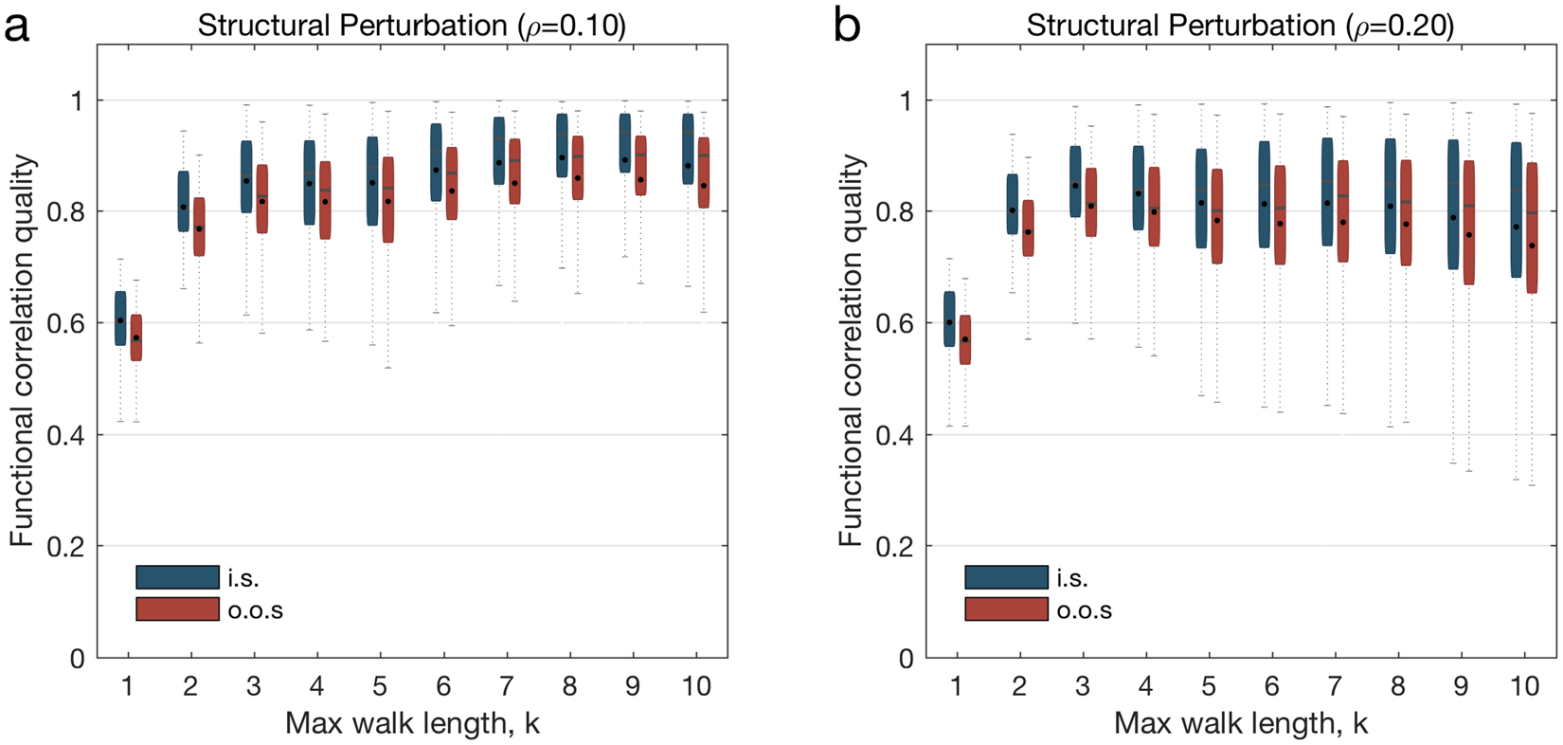
Robustness of the mapped functional connectivity to perturbations in the structural connectivity matrix for the Glasser-360 parcellation. In (**a**) and (**b**), we present the distribution of functional correlation quality of the mapped functional connectivity matrix as a function of the maximum walk length when the individual’s structural connectivity matrix has its entries perturbed by at most 10% (0.10) and 20% (0.20), respectively.

Finally, in order to assess the robustness of the individual spectral mapping, we compare the functional correlation quality ucorr $(\hat{F},\check{F})$ for all individuals and different values of parameter *ρ*, the results of which we present in Fig. 10. In particular, we consider the parameter *ρ *= {0.10,0.20} and generate perturbation matrices for each individual with entries obtained according to (10). Take the parameter *k* = 8. The out-of-sample functional correlation achieved was 0.9410 for the unperturbed case, whereas we achieve 0.8981 and 0.8165 for the perturbed case with *ρ *= 0.10 and *ρ *= 0.20, respectively. Therefore, these results support the fact that the proposed mapping presents considerable robustness with respect bounded perturbations in the structural matrix.

### Similarity of spectral characteristics across individuals

**Figure 11:**
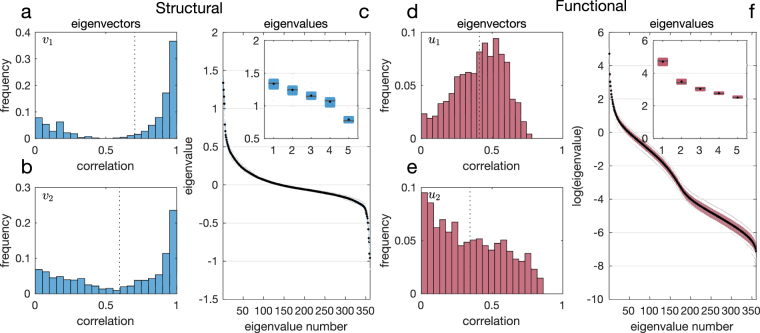
Spectral characteristics for structural and functional connectivity matrices (for all subjects). In (**a**,**b**) (respectively **d**,**e**), histograms of eigenvector correlations are displayed for the eigenvectors associated with the two largest eigenvalues of the structural (respectively, functional) matrices. Panel (c) (respectively, **f**) displays the distribution of ordered eigenvalues in the set of structural (respectively, functional) matrices, with the five largest eigenvalues magnified in the inset.

From a mathematical perspective, the effectiveness of the group spectral mapping method is, in part, explained by the similarity of spectral characteristics across subjects in the dataset. In particular, as we illustrate below, the eigenvalues of the functional (respectively, structural) matrix present a high level of similarity across individuals. Furthermore, the most relevant eigenvectors of these matrices (i.e., those associated with the largest eigenvalues) are also well aligned among individuals. To evaluate spectral similarities in the data, we start by plotting the eigenvalues of the structural matrix (in decreasing order) in Fig. 11c. In this figure, we include a box plot for the eigenvalues in the dataset. More precisely, for each eigenvalue number (i.e., the *i*-th eigenvalue number of each matrix, in decreasing order), we represent the average value, as well as the first and third quartile for the 44 values corresponding to the *i*-th eigenvalues of all the individuals in the dataset. We observe how the distribution of eigenvalues is very concentrated; in other words, the eigenvalues of the structural matrices in our dataset are very similar across individuals. Similarly, in Fig. 11f, we include a box plot for the eigenvalues of the functional matrices. We observe that, in this case, the eigenvalues are also similar across the dataset. We now shift our attention to the similarity between eigenvectors. In particular, we study the alignment among the first eigenvectors of the structural matrices (i.e., those eigenvectors associated to the largest eigenvalue) across individuals in the dataset. For each pair of individuals, we compute the correlation between their first eigenvectors. Since our dataset contains *l* = 44 individuals, we have $l(l-\mathrm{1)/2}=946$ possible pairs. In Fig. 11a, we plot a histogram for the values of all these correlations and observe that, in average, the first eigenvectors present a 70.4% correlation. We repeat this computation using the second eigenvectors of the structural matrices, and plot our results in Fig. 11b. Similarly, in Fig. 11d–e, we plot the histogram of correlations for the first and second eigenvectors of the functional matrices, respectively.

From a mathematical perspective, these spectral similarities allow us to find a high-quality ‘universal’ mapping able to map the adjacency matrix of structural brain graphs into functional connectivity matrices using tools from spectral graph theory. From a neurophysiological perspective, such similarities indicate that healthy human subjects display a similar organization of walks in structural graphs, as well as functional connectivity matrices.

### Comparison to null models

**Figure 12:**
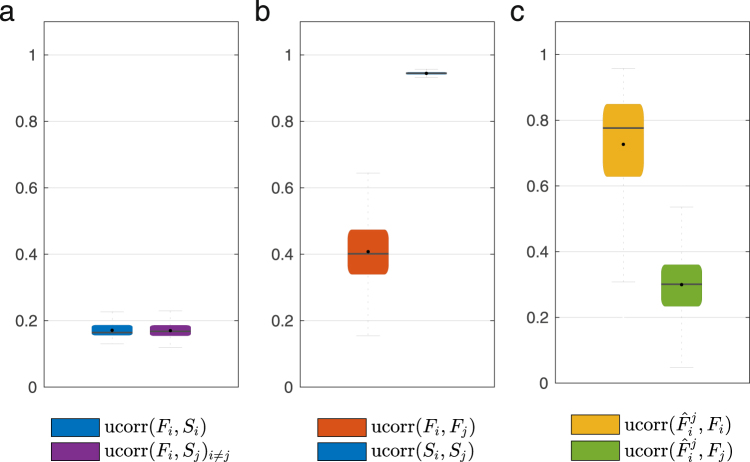
Null model analysis (for all subjects). We consider the parameter *k* = 8. Briefly, in (**a**) we present the inherent similarities across modalities, while in (**b**) we evaluate the similarities across subjects, for each modality. In addition, in (**c**) we consider the performance of individual spectral mapping when operating with shuffled data or parameters.

To gain critical understanding on the effectiveness of our method, we perform a series of tests considering several null models, using the function ucorr(*A*, *B*). We consider the parameter *k* = 8. Using this function, we first evaluate ucorr(*F*_*i*_, *S*_*i*_) for each individual *i* in the dataset. This correlation measures the inherent similarities between structural and functional modalities for the same subject. In Fig. 12a, we display in blue a box plot summarizing the distribution of correlations, which yields a mean (and standard deviation) of 0.1709(0202), respectively. Apart from similarities between structural and functional matrices for the same subject, we also measure inherent similarities for different subjects. In this direction, we evaluate ucorr(*F*_*i*_, *S*_*i*_) for all pairs (*i*, *j*) of individuals in the dataset. In Fig. 12a, we display in purple a box plot summarizing the distribution of correlations, which yields a mean (and a standard deviation) of 0.1699(0.0211). These numerical results suggest that the functional matrices *F*_*i*_ in our dataset are poorly correlated with the structural matrices *S*_*i*_; in fact, the average correlation between *F*_*i*_ and *S*_*i*_ is similar to the average correlation between *F*_*i*_ and *S*_*j*_ for $j\ne i$. Furthermore, we also analyze similarities among the functional (respectively, structural) matrices in the dataset by evaluating ucorr(*F*_*i*_, *F*_*j*_) (respectively, ucorr(*S*_*i*_, *S*_*j*_)) for all pairs (*i*, *j*) of individuals in the dataset. In Fig. 12b, we display box plots summarizing the distribution of correlations for the functional and structural modalities, whose means (and standard deviations) are 0.4077(0.0901) and 0.9445(0.0054), respectively. From these results, we observe that structural matrices present a significant level of correlation across individuals, while functional matrices present a lower average correlation.

In the above experiments, we have examined inherent similarities in the dataset by studying correlations between structural and functional matrices for the same, as well as different, individuals. In the following set of experiments, we evaluate the similarity between the ‘personalized’ functional mapping and functional matrices. As previously described, the ‘personalized’ functional mapping for the *i*-th individual in the dataset is trained using the pair of matrices ucorr(*S*_*i*_, *F*_*i*_) and characterized by the set of parameters *a*_*i*_ and *R*_*i*_. The input of this mapping is a structural matrix *S* and its output, denoted by ${\hat{F}}_{i}(S)$, is a functional matrix mapped from *S*. In the next experiment, we first compute the functional matrix mapped when the input is the *j*-th structural connectivity *S*_*j*_ using the ‘personalized’ mapping corresponding to the *i*-th individual. We denote this matrix as ${\hat{F}}_{i}^{j}={\hat{F}}_{i}({S}_{j})$. We then compare this matrix with *F*_*i*_ and *F*_*j*_ by computing ${\rm{ucorr}}\,({\hat{F}}_{i}^{j},{F}_{i})$ and ${\rm{ucorr}}({\hat{F}}_{i}^{j},{F}_{j})$ for all pairs of individuals (*i*, *j*) in the dataset. In Fig. 12c, we display in gray a box plot summarizing the distribution of ${\rm{ucorr}}\,({\hat{F}}_{i}^{j},{F}_{i})$, whose mean (and standard deviation) is 0.7267(0.1642). This relatively high value is, in part, explained by the inherent similarity among structural matrices of different subjects, as pointed out in the previous paragraph. Similarly, in Fig. 12c, we display in brown a box plot summarizing the distribution of ${\rm{ucorr}}\,({\hat{F}}_{i}^{j},{F}_{j})$ for all $i\ne j$, whose mean (and standard deviation) is 0.2996(0.0988). These values should be compared with ${\rm{ucorr}}\,({\hat{F}}_{i}^{i},{F}_{i})$, i.e., the correlation between the mapped and actual functional matrices for the *i*-th individual. As plotted in Fig. 3a, the average correlation in this case is much higher (at 0.9828(0.0779) for the in-sample case, and at 0.9092(0.0755) for the out-of-sample case). From these comparisons, it is possible to conclude that the spectral mapping method captures features that are specific to each individual’s structural and functional connectivity matrices, since such mapping is not reproducible (on average) by swapping either the matrices or the parameters associated with the mapping.

## Discussion

In analyzing the methodological advantages and disadvantages of our proposed method, the following observations can be made. The individual spectral mapping is mathematically assured to asymptotically approximate the functional matrix, as the parameter controlling the maximum walk length is increased. Therefore, it provides a first-principles mapping between the structural and functional connectivity matrices, which is interpretable in terms of a weighted combination of walks of different lengths and the alignment of structural and functional eigenmodes. As a potential disadvantage, a critique might be raised on the number of degrees of freedom introduced by the rotation matrix. To address that concern, we have shown in subsection ‘Stability of spectral mapping’ that the number of degrees of freedom can be controlled by using rank-constrained rotation matrices, with little relative impact in the approximation quality, as shown in Fig. 9. In contrast, the group spectral mapping enables a novel approach to analyze common spectral characteristics between groups of individuals, with a relatively lower number of degrees of freedom, but a more challenging computational approach. As a result, the solution to the group mapping problem relies on the use of advanced numerical optimization algorithms to handle the constraints imposed by the common rotation matrix, as described in Section 3.2 of the SI.

Our results have important implications for cognitive neuroscience. First, it is remarkable that walks of length up to *k* = 3 offer the most contribution for mapping accuracy, even across structural brain networks constructed from different spatial resolutions. This surprisingly low value of *k* suggests that relatively parsimonious polysynaptic connections impose critical constraints on brain dynamics and observed functional connectivity. The disproportionate contribution of short walks (of length up to 3) to the functional mapping may be due to energy considerations: it is intuitively plausible that processing information along longer walks may require more energy than processing information along shorter walks. An alternative explanation could lie in the temporal constraints imposed by our environment: over evolutionary time scales, the time it takes an organism to respond to threats or opportunities is negatively correlated with the organism’s reproductive success. Assuming longer walks require more time and more energy; it is then reasonable that relatively short walks in the structural graph can map the observed functional dynamics. However, this line of argument also begs the question of why the functional connectivity matrices cannot be mapped with high accuracy using only walks of length *k* = 1 or *k* = 2. To address this question, it is important to note that walks of increasing length offer a greater dimensionality to the dynamic range of the system. Systems that only utilize structural walks of length *k* = 1 necessarily have an impoverished ensemble of possible states in comparison to those that utilize structural walks of longer lengths. Thus, it is intuitively plausible that the mapping accuracy obtained from *k* = 3 is an indirect indication of a careful balance between the competing requirements for a broad dynamic range and an energetically and temporally efficient system.

Finally, it is important to note that the methods we develop here are more generally applicable to other problems in which one wishes to map one set of matrices from another set of matrices. In the context of neuroimaging, we could use these same tools to ask how structural graphs prior to an injury relate to structural graphs after an injury. We could also ask whether and how functional connectivity matrices change over time, either during learning or as a function of normal aging. It will be interesting in future to determine whether features of the rotation matrix (the mapping from structure to function) are related to individual differences in cognitive abilities in healthy subjects, symptomatology in diseased cohorts, or genetic variability.

## Electronic supplementary material

Supplementary Information
