# Supplementary material for: Spectral mapping of brain functional connectivity from diffusion imaging
Source: Sci Rep. 2018 Jan 23;8:1411. doi: 10.1038/s41598-017-18769-x (PMC5780460; doi:10.1038/s41598-017-18769-x)
Supplement: Supplementary file 1 — Supplementary Information [file 41598_2017_18769_MOESM1_ESM.pdf]

Supplementary Information for "Spectral mapping of brain functional connectivity from diffusion imaging"

Cassiano O. Becker[1], Sérgio Pequito[2], George J. Pappas[1],
Michael B. Miller[3], Scott T. Grafton[3], Danielle S. Bassett[1,4] and Victor M. Preciado[1]

[1] Department of Electrical and Systems Engineering, University of Pennsylvania, Philadelphia, USA
[2] Department of Industrial and Systems Engineering, Rensselaer Polytechnic Institute, Troy, USA
[3] Department of Psychological and Brain Sciences, University of California at Santa Barbara, USA
[4] Department of Bioengineering, University of Pennsylvania, Philadelphia, USA

December 26, 2017

Contents

1 Overview **2**

2 Notation **2**

3 Spectral mapping: mathematical description **3**
3.1 Individual spectral mapping problem . . . 4
3.2 Group spectral mapping problem . . . 8

4 Supplementary methods: AAL-parcellated dataset **11**
4.1 Study subjects . . . 11
4.2 MRI data acquisition . . . 11
4.3 Connectivity estimates . . . 12
4.3.1 Functional connectivity . . . 12
4.3.2 Structural connectivity . . . 13
4.4 Cortical parcellation . . . 14
4.4.1 Brain region masks . . . 14
4.4.2 Atlases and upsampling procedure . . . 14
4.5 Connectivity matrices . . . 14

5 Supplementary results: AAL-parcellated dataset **15**
5.1 Recovered connectivity matrices . . . 15
5.2 Perturbation analysis . . . 15

6 Comparison with baseline methods **16**
6.1 DKHM . . . 18
6.2 Taylor Approximation . . . 21

1 Overview

This document provides additional information supporting the findings presented in the main article. Formal mathematical derivations of the spectral mapping methods are provided and summarized in the form of algorithmic descriptions, where the input and output assumptions are emphasized, in Section 3. The method proposed to solve the individual spectral mapping problem is presented in Section 3.1, and the one to solve the group spectral mapping problem in Section 3.2. Next, in Section 4, we describe the acquisition and pre-processing of a dataset following the Automated Anatomical Labeling parcellation (AAL) [30]. In Section 5, we report the results of the individual spectral mapping when the AAL-parcellated dataset is matrices are considered. Specifically, in Section 5.1, we present mapping results obtained using the individual and group functional mapping, and, in Section 5.2, we show that the individual functional mapping is robust with respect to perturbations in the structural connectivity matrix. In Section 6, we compare our proposed approach, i.e., the individual spectral mapping, with up-to-date methods among those found in the literature. In particular, we first perform a comparison with a method that invokes the description of the functional connectivity matrix as a combination of graph theoretical metrics in Section 6.1. Alternatively, in Section 6.2, we compare our method with a local approximation of a possible non-linear mapping that is described in terms of a truncated Taylor approximation.

2 Notation

We denote by x a column vector in $\mathbb{R}^n$ and by x_i its i-th entry. Bold capital letters are reserved for matrices, for instance $X, Y \in \mathbb{R}^{m\times n}$, where $[X]_{ij}$ (or, equivalently, x_{ij}) denotes the entry in the i-th row and j-th column of X. The transpose of a matrix is written as $X^\intercal$, i.e., $[X^\intercal]_{ij} = [X]_{ji}$. The $n \times n$ identity matrix is denoted by $I_n = \mathrm{diag}(1_n)$, where $\mathrm{diag}(x)$ is a diagonal matrix having the elements of x in its diagonal, and 1_n is the $n \times 1$ vector of ones. The square matrix $X \in \mathbb{R}^{n\times n}$ is said to be symmetric if $X = X^\intercal$. The inner product of two vectors is defined by $\langle x, y\rangle = x^\intercal y = \sum_{i=1}^n x_i y_i$, and the Euclidean norm of a vector is given by $\|x\|_2 = (x^\intercal x)^{\frac{1}{2}}$. The inner product of two square matrices is given by $\langle X, Y\rangle = \sum_{i=1}^n \sum_{j=1}^n x_{ij} y_{ij} = \mathrm{tr}\,(X^\intercal Y)$ with $\mathrm{tr}\,(A) = \sum_{i=1}^n a_{ii}$. The Frobenius norm of a matrix is defined as $\|X\|_{\mathcal{F}} = (\sum_{i=1}^m \sum_{j=1}^n x_{ij}^2)^{\frac{1}{2}}$, yielding $\|X\|_{\mathcal{F}}^2 = \mathrm{tr}\,(X^\intercal X)$. A square matrix X is said to be orthogonal if $X^\intercal X = I_n$. Given a vector x, we define its corresponding average vector by $\bar{x} = \frac{1}{n}(\sum_{i=1}^n x_i)\, 1_n$. Similarly, the $m \times n$ average matrix of $X \in \mathbb{R}^{m\times n}$ is defined as $\overline{X} = \frac{1}{mn}(\sum_{i=1}^m \sum_{j=1}^n [X]_{ij})\, 1_m\, 1_n^\intercal$.

The columns of a matrix $X \in \mathbb{R}^{m\times n}$, given by

$$X = [\, x_1 \,|\, x_2 \,|\, \cdots \,|\, x_n \,],$$

can be vertically concatenated using the so-called *vectorization operator*, denoted by $\mathrm{vec}(X)$; more specifically, we have

$$\mathrm{vec}(X) = [x_1^\intercal, \ldots, x_n^\intercal]^\intercal = [\, x_{11}, \ldots, x_{m1}, \ \ldots\ , \ x_{1n}, \ldots, x_{mn} \,]^\intercal ,$$

which allows us to write $\|X\|_{\mathcal{F}} = \|\text{vec}(X)\|_2$. Similarly, for a symmetric matrix $A \in \mathbb{R}^{n\times n}$, i.e.,

$$A = \begin{bmatrix} \star & a_{12} & a_{13} & \cdots & a_{1n} \\ & \star & a_{23} & & \vdots \\ & & \star & \ddots & \vdots \\ & & & \star & a_{(n-1)n} \\ & & & & \star \end{bmatrix},$$

the *upper-triangular vectorization operator*, denoted by $\text{uvec}(A)$, returns a vector containing the elements above the main diagonal as follows:

$$\text{uvec}(A) = \begin{bmatrix} a_{12}, \, a_{13}, \, a_{23}, \, a_{14}, \, a_{24}, \, a_{34}, \ldots, a_{1n}, \ldots, a_{(n-1)n} \end{bmatrix}^\intercal .$$

Recall that the correlation coefficient between two vectors $x, y \in \mathbb{R}^n$ is defined as

$$\text{corr}(x, y) = \frac{(x - \bar{x})^\intercal (y - \bar{y})}{\|x - \bar{x}\|_2 \, \|y - \bar{y}\|_2}.$$

Subsequently, given two $n \times n$ real matrices X and Y, we define *matrix correlation* as

$$\text{mcorr}(X, Y) = \frac{\text{tr}\left((X - \overline{X})^\intercal (Y - \overline{Y})\right)}{\|X - \overline{X}\|_{\mathcal{F}} \, \|Y - \overline{Y}\|_{\mathcal{F}}}.$$

Further, using the vectorization operator, we have that $\text{mcorr}(X, Y) = \text{corr}(\,\text{vec}(X),\, \text{vec}(Y)\,)$.

In addition, given two symmetric matrices X and Y, we define the *upper (off-diagonal) matrix correlation* as

$$\text{ucorr}(X, Y) = \text{corr}(\,\text{uvec}(X),\, \text{uvec}(Y)\,),$$

which is adopted as a measure of the similarity between matrices in the experimental evaluations of this paper, and is referred to as *functional correlation quality* when applied to functional connectivity matrices.

A vector x is referred to as an *eigenvector* of the (symmetric) matrix A when there exists a (real) scalar λ such that the equality $Ax = \lambda x$ is satisfied. If λ satisfies this equality for a given x, then we say that λ is the *eigenvalue* of A associated with the eigenvector x. Similarly, we say that the eigenvector x is associated with the eigenvalue λ; hence, refer to (λ, x) as an eigenvalue-eigenvector pair. In addition, we assume that the eigenvalues are sorted, i.e., $\lambda_1 \leq \ldots \leq \lambda_n$; subsequently, this induces an order over the eigenvalue-eigenvector pairs. In what follows, we refer to the i-th eigenvector as the eigenvector associated with the λ_i eigenvalue.

3 Spectral mapping: mathematical description

We now describe in detail the functional mapping proposed in the main article. The overall goal is to determine a mapping to estimate the functional connectivity matrix from the structural connectivity, as illustrated in Fig. S1. We refer to this problem as the *mapping problem*. First, we consider a mapping problem in which the objective is to find a mapping between the structural and the functional connectivity matrices of a single individual, as illustrated in Fig. S2. We refer to this problem as *individual spectral mapping*, whose solution is described in Section 3.1. An alternative, and closely related, problem can be stated by considering

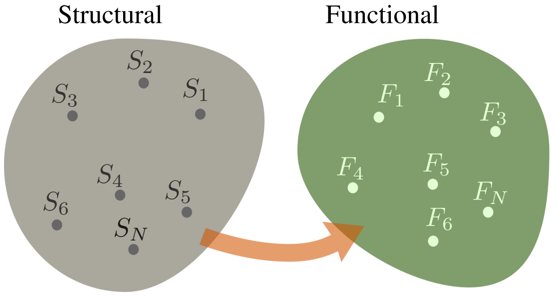

Fig. S1 Schematics of the mapping problem. The set of points in the structural set (left) represents structural connectivity matrices for the individuals in the dataset, while points in the functional set (right) represent their functional matrices. Our goal is to determine a mapping between these two sets.

the mapping between the average structural matrix and the average functional matrix, where the averages are computed over the whole set of individuals in the dataset, as illustrated in Fig. S3. Additionally, in Section 3.2, we consider the so-called *group spectral mapping problem* in which we aim towards finding a common mapping able to map the functional connectivity matrix of any individual in a group from his/her structural connectivity matrix, as illustrated in Fig. S4 and Fig. S5. In other words, we aim to finding a single mapping, defined by a common set of parameters shared by a set of individuals.

3.1 Individual spectral mapping problem

In this particular mapping problem, we are given two $n \times n$ symmetric matrices, S and F, corresponding to the structural and functional connectivity matrices of an individual. Our objective is to build a mapping $\hat{F}$, as a function of S, of the functional matrix F. This mapping is constructed as follows: in the first stage, we consider a linear combination of $k+1$ powers of the matrix S, up to the k-th term (see Fig. 1 in the main article). In the second stage, we apply a rotation between the structural and functional reference frames (see details in the Rotation Matrix section in the main manuscript). The rotation acts as change of coordinates, which is achieved by applying an orthogonal matrix R as a similarity transformation. Therefore, the spectral mapping can be formally described as follows:

$$\hat{F} = R\, f(S) R^{\intercal}, \quad \text{where} \quad f(S) = \sum_{i=0}^{k} a_i\, S^i. \tag{1}$$

As described in the **Materials and Methods** section of the main paper, we find the parameters $\{a_i\}_{i=0}^{k}$ and R by solving the following optimization problem:

$$\begin{array}{ll} \underset{\{a_i\}_{i=0}^{k}, R}{\text{minimize}} & \left\| F - R\left(\sum_{i=0}^{k} a_i\, S^i\right) R^{\intercal} \right\|_{\mathcal{F}}^{2} \\ \text{subject to} & RR^{\intercal} = R^{\intercal}R = I_n. \end{array} \tag{2}$$

In fact, we would also need to include the extra constraint $\det(R) = 1$, but due to the quadratic nature of the objective function, we can waive this constraint. In other words, we notice that the constraint $\det(R) = 1$ can be omitted by reasoning as follows. First, observe that $|\det(R)| = 1$ is guaranteed by the constraint $R^{\intercal}R = I$, which, by the determinant product rule, gives $1 = \det(I) = \det(R^{\intercal}R) = \det(R^{\intercal})\det(R) = (\det(R))^2$, implying that $\det(R) = \pm 1$. Now, suppose that R_+ and R_- are two solutions of (21), where $\det(R_+) = 1$ and $\det(R_-) = -1$, since both satisfy the constrains considered. It readily follows that R_+ is a rotation matrix that solves the problem proposed in (1). Alternatively, notice that although R_- is not a rotation matrix

(since $\det(R_-) = -1$), when applied in the mapping given in (1), it yields the same $\hat{F}$ as the one obtained when using R_+. Furthermore, notice that from R_- we can obtain a solution to (20) by considering $R' = (-1)R_-$. Specifically, notice that R' is a solution to (2) since now $\det(R') = 1$, i.e., it is a rotation matrix.

In what follows, we propose a solution to this problem using spectral methods. Let us consider the eigenvalue decomposition of S, which is given by $S = V\Lambda V^{-1} = V\Lambda V^{\intercal}$, where $\Lambda = \mathrm{diag}(\lambda_1, \ldots, \lambda_n)$ is the diagonal matrix containing the sorted eigenvalues of S and the columns of the matrix $V = [v_1|v_2|\ldots|v_n]$ are the sorted associated eigenvectors of S. Hence, $f(S)$ can be rewritten as

$$f(S) = \sum_{i=0}^{k} a_i \, (V\Lambda V^{\intercal})^i = \sum_{i=0}^{k} a_i \, V\Lambda^i V^{\intercal} = V \left(\sum_{i=0}^{k} a_i \, \mathrm{diag}(\lambda^i) \right) V^{\intercal}, \tag{3}$$

where $\lambda^i = [\lambda_1^i, , \ldots, \lambda_n^i]^{\intercal}$. Therefore, (2) can be rewritten as:

$$\begin{array}{ll} \underset{\{a_i\}_{i=0}^{k}, R}{\text{minimize}} & \left\| F - RV \left(\sum_{i=0}^{k} a_i \, \mathrm{diag}(\lambda^i) \right) V^{\intercal} R^{\intercal} \right\|_{\mathcal{F}}^2 \\ \text{subject to} & RR^{\intercal} = R^{\intercal}R = I_n. \end{array} \tag{4}$$

Notwithstanding, the problem in (4), defined jointly over the variables $\{a_i\}_{i=0}^{k}$ and R is computationally challenging [1]. We propose the following decomposition approach. Let us denote the eigenvalues and eigenvectors of F by $\{\varphi_i\}_{i=1}^{n}$ and $\{u_i\}_{i=1}^{n}$, respectively. Define the vector of eigenvalues $\varphi = (\varphi_1, \ldots, \varphi_n)^{\intercal}$ and the matrix of eigenvectors $U = [u_1|u_2|\cdots|u_n]$. The eigen-decomposition of F is given by

$$F = U \, \Phi \, U^{\intercal},$$

where $\Phi = \mathrm{diag}(\varphi)$. Using this eigen-decomposition, the objective function in (4) can be rewritten as

$$\left\| U \mathrm{diag}(\varphi) U^{\intercal} - RV \left(\sum_{i=0}^{k} a_i \mathrm{diag}(\lambda^i) \right) V^{\intercal} R^{\intercal} \right\|_{\mathcal{F}}^2 . \tag{5}$$

Ideally, if we could impose the constraints $U = RV$ and $\mathrm{diag}\,(\varphi) = \left(\sum_{i=0}^{k} a_i \mathrm{diag}\,(\lambda^i) \right)$, the Frobenius norm in (5) would be zero. In general, it is not possible to achieve the equality $\mathrm{diag}\,(\varphi) = \left(\sum_{i=0}^{k} a_i \mathrm{diag}\,(\lambda^i) \right)$ for a given value of $k < n$. Therefore, we propose to impose the constraint $U = RV$ (which can always be done by forcing $R = UV^{-1} = UV^{\intercal}$), and find the set of

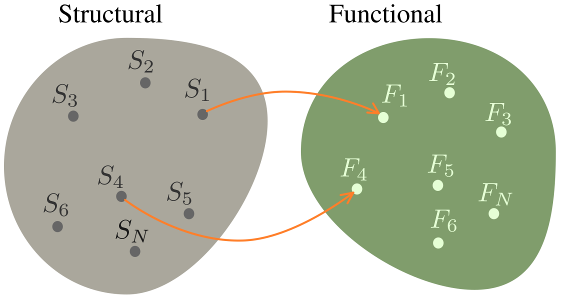

Fig. S2 The mapping problem for single individuals. Functional connectivity is approximated for each subject based on its structural and functional connectivity matrices.

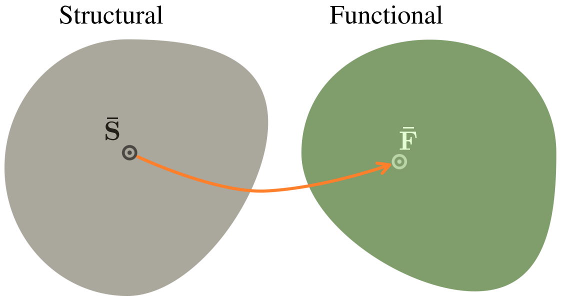

Fig. S3 Mapping problem for the representative individual. Matrices from all individuals in the dataset are averaged element-wise to form the representative structural and representative functional connectivity matrices.

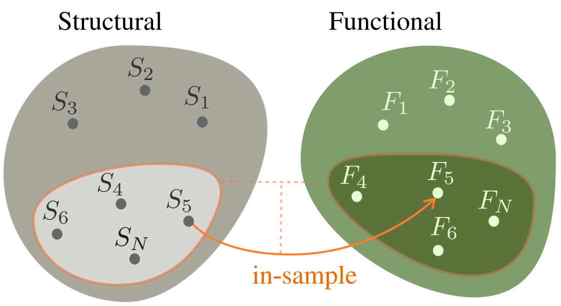

Fig. S4 The group spectral mapping problem (in-sample). Mapping parameters are estimated from a training set consisting of a collection of structural and functional matrices associated with a subset of individuals. Subsequently, this mapping can be used to assess an in-sample individual's functional from its structural connectivity matrix.

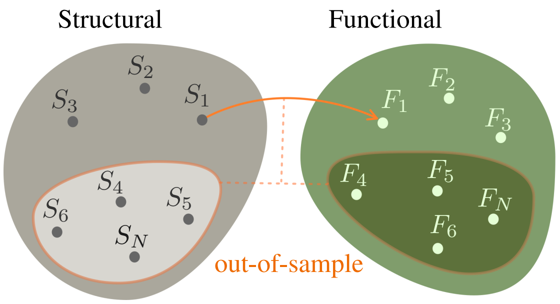

Fig. S5 The group spectral mapping problem (out-of-sample). After the spectral mapping has been determined with the in-sample elements, as explained in Fig. S4, the same mapping is used to assess an out-of-sample individual's functional matrix estimate.

values $\{a_i\}_{i=1}^k$ by solving the following optimization

$$\underset{\{a_i\}_{i=0}^k}{\text{minimize}} \quad \left\| \text{diag}(\varphi) - \sum_{i=0}^{k} a_i \, \text{diag}(\lambda^i) \right\|_{\mathcal{F}}^2 ,$$

or, equivalently,

$$\underset{\{a_i\}_{i=0}^k}{\text{minimize}} \quad \left\| \varphi - \sum_{i=0}^{k} a_i \lambda^i \right\|_2^2 . \tag{6}$$

This is a well-known least squares approximation problem, whose minimizer is given by

$$a^\star = (L^\intercal L)^{-1} L^\intercal \varphi, \tag{7}$$

where L is the Vandermonde matrix described as follows

$$L = \begin{bmatrix} 1 & \lambda_1 & \lambda_1^2 & \dots & \lambda_1^k \\ 1 & \lambda_2 & \lambda_2^2 & \dots & \lambda_2^k \\ \vdots & \vdots & \vdots & & \vdots \\ 1 & \lambda_n & \lambda_n^2 & \dots & \lambda_n^k \end{bmatrix} . \tag{8}$$

Therefore, the individual mapping $\hat{F}$ can be obtained as follows

$$\hat{F} = RV \left(\sum_{i=0}^{k} a_i^\star \text{diag}(\lambda^i) \right) V^\intercal R^\intercal = U \left(\sum_{i=0}^{k} a_i^\star \text{diag}(\lambda^i) \right) U^\intercal ,$$

where in the last equality we use the identity $R = UV^\intercal$.

In what follows, we analyze the error incurred by our approximation method. Define the matrices $\hat{\Phi} = \sum_{i=0}^{k} a_i^\star \, \text{diag}(\lambda^i)$ and $\Delta_\Phi = \hat{\Phi} - \Phi$. Then, the objective function in (4) evaluated at our approximated solution (i.e., $a^\star = (L^\intercal L)^{-1} L^\intercal \varphi$ and $R = UV^\intercal$)

is given by

$$\left\| F - RV \left(\sum_{i=0}^{k} a_i^\star \operatorname{diag}(\lambda^i) \right) V^\intercal R^\intercal \right\|_{\mathcal{F}}^2 = \left\| F - RV\hat{\Phi}V^\intercal R^\intercal \right\|_{\mathcal{F}}^2 = \left\| U\Phi U^\intercal - RV\left(\Phi^2 + \Delta_\Phi\right) V^\intercal R^\intercal \right\|_{\mathcal{F}}^2 .$$

Therefore, since $R = UV^\intercal$, we obtain

$$\left\| U\Phi U^\intercal - U\left(\Phi + \Delta_\Phi\right) U^\intercal \right\|_{\mathcal{F}}^2 = \left\| U\Delta_\Phi U^\intercal \right\|_{\mathcal{F}}^2 = \left\| \Delta_\Phi \right\|_{\mathcal{F}}^2 , \tag{9}$$

where the last equality follows from the fact that pre- and post-multiplying a matrix by an orthonormal matrix does not change its Frobenius norm. Notice that

$$\left\| \Delta_\Phi \right\|_{\mathcal{F}}^2 = \left\| \hat{\Phi} - \Phi \right\|_{\mathcal{F}}^2 = \left\| \sum_{i=0}^{k} a_i^\star \operatorname{diag}(\lambda^i) - \operatorname{diag}(\varphi) \right\|_{\mathcal{F}}^2 = \left\| \sum_{i=0}^{k} a_i^\star \lambda^i - \varphi \right\|_2^2 ,$$

which is equal to the cost function in (6). This cost monotonically decreases as the parameter k in (6) increases; therefore, the approximation error $\left\| \Delta_\Phi \right\|_{\mathcal{F}}^2$ decreases as k increases.

Computational procedure

For convenience, we summarize in **Algorithm 1** the procedure described in Section 3.1 to obtain the parameters for the individual spectral mapping.

Algorithm 1
Estimation of parameters and functional connectivity matrix for the individual spectral mapping.

Input :
structural connectivity matrix S;
functional connectivity matrix F;
maximum order k of the functional mapping.

Procedure :
1: *compute* the matrix of eigenvectors V and the vector of eigenvalues λ of S;
2: *compute* the matrix of eigenvectors U and the vector of eigenvalues φ of F;
3: *set* $a^\star = (L^\intercal L)^{-1} L^\intercal \varphi$, where L is the Vandermonde matrix defined in (8);
4: *set* $R = UV^\intercal$.

Output :
functional matrix estimate $\hat{F} = R \left(\sum_{i=0}^{k} a_i^\star S^i \right) R^\intercal$.

Rank-constrained solution

Following the analysis of the stability of spectral mapping proposed in the main manuscript, we now describe the approach considered for the rank-constrained solution. Specifically, since an $n \times n$ rotation matrix R has $n(n-1)/2$ degrees of freedom [12], the solution to (2) may result in overfitting due to the large number of parameters in the mapping. To test that we are not overfitting, we consider a version of the optimization problem in (2) in which we constrain the rank of R to be equal to a given integer $m \in \{1, \ldots, n\}$. In order to reduce the rank of the rotation matrix, we consider low-rank approximations of S and F, as follows. Define the $m \times m$ matrices $\Lambda_{(m)} = \operatorname{diag}(\lambda_1, \ldots, \lambda_m)$ and $\Phi_{(m)} = \operatorname{diag}(\phi_1, \ldots, \phi_m)$ containing the top m eigenvalues of S

and F, respectively. Correspondingly, define the $n \times m$ matrices $V_{(m)} = [\, v_1 \,|\, v_2 \,|\, \ldots \,|\, v_m \,]$ and $U_{(m)} = [\, u_1 \,|\, u_2 \,|\, \ldots \,|\, u_m \,]$ containing the top m eigenvectors of S and F, respectively. Then, we have that the best m-rank approximations of S and F are given by $S_{(m)} = V_{(m)} \Lambda_{(m)} V_{(m)}^{\mathsf{T}}$ and $F_{(m)} = U_{(m)} \Phi_{(m)} U_{(m)}^{\mathsf{T}}$, respectively. Therefore, the rotation matrix aligning these m-rank approximations is given by $R_{(m)} = U_{(m)} V_{(m)}^{\mathsf{T}}$. Notice that $R_{(m)}$ is itself an m-rank matrix.

3.2 Group spectral mapping problem

In this section, we consider a group of N individuals whose structural and functional matrices are given by the set of pairs $\{(S_j, F_j)\}_{j=1}^{N}$. In this mapping problem, our objective is to find a *common* mapping able to generate an approximation of F_j from S_j, while addressing the following question: can a mapping be obtained in terms of *common functional eigenmodes* across the different subjects? In other words, we seek to determine the common resting state functional patterns among the subjects. Towards this goal, we propose to determine a mapping $\hat{F}_j = Q\, g(S_j) Q$, where $g(S_j) = \sum_{r=0}^{k} c_r \mathrm{diag}(\lambda_j^r)$, with $\lambda_j^r = (\lambda_1^r(S_j), \ldots, \lambda_n^r(S_j))$ and Q is an orthogonal matrix encapsulating the functional eigenvectors. In particular, notice that the structural eigenvectors of a specific subject are not considered, since we aim to find a common set of functional eigenmodes.

Also, let us denote the eigenvectors of the $n \times n$ structural matrix S_j by $\{v_i(S_j)\}_{i=1}^{n}$, and the matrix of eigenvectors by $V_j = [\, v_1(S_j) \,|\, v_2(S_j) \,|\, \cdots \,|\, v_n(S_j) \,]$. Consider the eigen-decomposition of the structural matrix of individual j, which is given by

$$S_j = V_j \mathrm{diag}(\lambda_j) V_j^{\mathsf{T}}. \tag{10}$$

Also, let us denote the eigenvalues and eigenvectors of F_j by $\{\varphi_i(F_j)\}_{i=1}^{n}$ and $\{u_i(F_j)\}_{i=1}^{n}$, respectively. Define the vector of eigenvalues $\varphi_j = (\varphi_1(F_j), \ldots, \varphi_n(F_j))^{\mathsf{T}}$ and the matrix of eigenvectors $U_j = [\, u_1(F_j) \,|\, u_2(F_j) \,|\, \cdots \,|\, u_n(F_j) \,]$. The eigen-decomposition of F_j is therefore given by

$$F_j = U_j \Phi_j U_j^{\mathsf{T}}, \tag{11}$$

where $\Phi_j = \mathrm{diag}(\varphi_j)$.

Therefore, the parameters $\{c_r\}_{r=0}^{k}$ and Q are the solution to the following optimization problem:

$$\begin{aligned} \underset{\{c_r\}_{r=0}^{k}, Q}{\text{minimize}} \quad & \sum_{j=1}^{N} \left\| F_j - Q \left(\sum_{r=0}^{k} c_r \, \mathrm{diag}(\lambda_j^r) \right) Q^{\mathsf{T}} \right\|_{\mathcal{F}}^{2} \\ \text{subject to} \quad & QQ^{\mathsf{T}} = Q^{\mathsf{T}} Q = I_n \,. \end{aligned} \tag{12}$$

Assume that $(\{c_r^\star\}_{r=0}^{k}, Q^\star)$ is the optimal solution to (12). Then, the $n \times n$ matrix $Q^\star = [\, q_1^\star \,|\, \ldots \,|\, q_n^\star \,]$ is called the matrix of common eigenmodes, where the columns $q_i^\star$ are the common eigenmodes. Notice that determining the optimal solution to the problem in (12) is computationally hard [1]. Therefore, we propose a two-step approximation strategy, which we describe in detail below. In short, in the first step of this approximation, we assume that $\{c_r^\star\}_{r=0}^{k}$ is given, and we find an $n \times n$ rotation matrix $Q^\star$ using manifold optimization methods, as described in the next paragraph. In the second step, once we have found $Q^\star$, we determine $\{c_r^\star\}_{r=0}^{k}$. In what follows, we describe each one of these two steps in more detail.

We now describe the first step of our approximation strategy. Let us assume that $\{c_r^\star\}_{r=0}^{k}$ is known, and define $\Phi_j^\star = \sum_{r=0}^{k} c_r^\star \, \mathrm{diag}(\lambda_j^i)$.

Then, the optimization problem in (12) reduces to determining the solution $Q^\star$ to the following optimization problem

$$\begin{aligned} \underset{Q}{\text{minimize}} \quad & \sum_{j=1}^{N} \left\| F_j - Q\Phi_j^\star Q^\intercal \right\|_{\mathcal{F}}^2 \\ \text{subject to} \quad & QQ^\intercal = Q^\intercal Q = I_n \, . \end{aligned} \tag{13}$$

The objective function in (13) can be simplified using the following equality $\|X\|_{\mathcal{F}}^2 = \operatorname{tr}(X^\intercal X)$. Further, for any matrices A, B and C with appropriate dimensions, we have $\operatorname{tr}(A+B) = \operatorname{tr}(A) + \operatorname{tr}(B)$ and $\operatorname{tr}(ABC) = \operatorname{tr}(CAB)$. Therefore, we can rewrite the objective in (13) as

$$\sum_{j=1}^{N} \left\| F_j - Q\,\Phi_j^\star Q^\intercal \right\|_{\mathcal{F}}^2 = \sum_{j=1}^{N} \operatorname{tr}\left(F_j^\intercal F_j\right) + \operatorname{tr}\left((\Phi_j^\star)^\intercal \Phi_j^\star\right) - 2\operatorname{tr}\left(F_j^\intercal Q\,\Phi_j^\star Q^\intercal\right). \tag{14}$$

We note in (14) that the only part depending on the optimization variable Q is the third term on the right-hand side. Therefore, $Q^\star$ is also the solution of the following optimization problem

$$\begin{aligned} \underset{Q}{\text{minimize}} \quad & -\operatorname{tr}\left(\sum_{j=1}^{N} F_j^\intercal Q\,\Phi_j^\star\, Q^\intercal\right) \\ \text{subject to} \quad & QQ^\intercal = Q^\intercal Q = I_n \, . \end{aligned} \tag{15}$$

There is no known closed-form solution to (15), and we adopt the iterative procedure described in [1, Chapter 7] to find an approximate solution. We denote by $\mathcal{L}$ the iterative mapping in this procedure. The theory and algorithms supporting this method are fully described in [1, 9] and a software package is provided in [6], which we used in our experiments.

Remark 1. *To evaluate the optimality of the solution obtained, one can first find a lower bound $q^\star$ of the optimal solution to (15) via the associated dual formulation of the optimization problem, presented in [7]. One can then measure the difference between the lower bound $q^\star$ and the objective function value $p(Q^{(r)})$, where $p : \mathbb{R}^{n\times n} \to \mathbb{R}$ is the objective function in* (15) *and $Q^{(r)}$ is the r-th iteration in the iterative procedure. This difference is known as the* optimality gap. *In particular, the optimality gap at iteration r can be used to provide a stopping criterion for the iterative procedure. Further, if this gap is equal to zero, the optimal solution to the problem in* (15) *has been determined.* ○

In the second step of our procedure, we assume that $Q^\star$ is the matrix obtained in the first step of our procedure. Under this assumption, the problem in (12) is reduced to the following optimization:

$$\underset{\{c_r\}_{r=0}^{k}}{\text{minimize}} \quad \sum_{j=1}^{N} \left\| F_j - Q^\star \left(\sum_{r=0}^{k} c_r \operatorname{diag}(\lambda_j^r)\right) (Q^\star)^\intercal \right\|_{\mathcal{F}}^2 , \tag{16}$$

and denote its solution by $\{c_r^\star\}_{r=0}^{k}$. This objective function can be rewritten as

$$\sum_{j=1}^{N} \left\| U_j \Phi U_j^\intercal - Q^\star \left(\sum_{r=0}^{k} c_r \operatorname{diag}(\lambda_j^r)\right) (Q^\star)^\intercal \right\|_{\mathcal{F}}^2 ,$$

where we have used the eigen-decomposition of F_j. Following the reasoning proposed in the individual spectral mapping, we

approximate the optimal values $\{c_r^\star\}_{r=0}^k$ by assuming $Q^\star = U_j$ for all j. Under this assumption, the cost function in (16) becomes

$$\sum_{j=1}^{N} \left\| \Phi - \left(\sum_{r=0}^{k} c_r \operatorname{diag}(\lambda_j^r) \right) \right\|_{\mathcal{F}}^2 ,$$

where we have used the fact that the Frobenius norm is invariant under orthonormal similarity transformations. Therefore, $\{c_r^\star\}_{r=0}^k$ can be determined by solving the following optimization problem

$$\underset{\{c_r\}_{r=0}^k}{\text{minimize}} \quad \sum_{j=1}^{N} \left\| \varphi_j - \sum_{r=0}^{k} c_r \lambda_j^r \right\|_2^2 . \tag{17}$$

The solution to this problem can be stated in matrix form, as follows:

$$c^\star = (L_v^\intercal L_v)^{-1} L_v^\intercal \varphi_v, \tag{18}$$

where

$$L_v = \begin{bmatrix} 1_n & \lambda_1 & \lambda_1^2 & \dots & \lambda_1^k \\ 1_n & \lambda_2 & \lambda_2^2 & \dots & \lambda_2^k \\ \vdots & \vdots & \vdots & & \vdots \\ 1_n & \lambda_N & \lambda_N^2 & \dots & \lambda_N^k \end{bmatrix} \text{ and } \varphi_v = \begin{bmatrix} \varphi_1 \\ \varphi_2 \\ \vdots \\ \varphi_N \end{bmatrix} . \tag{19}$$

Therefore, the mapping of each functional matrix F_j is given by

$$\hat{F}_j = Q^\star \left(\sum_{r=0}^{k} c_r^\star \operatorname{diag}(\lambda_j^r) \right) (Q^\star)^\intercal ,$$

where $Q^\star$ and $\{c_r^\star\}_{r=0}^k$ are the *common* parameters obtained from (15) and (18), respectively.

Computational procedure

For convenience, we summarize the procedure proposed to solve the group spectral mapping problem in **Algorithm 2**.

Algorithm 2 Estimation of parameters and functional connectivity matrices for the group spectral mapping.

Input :
collection of pairs $\{(S_j, F_j)\}_{j=1}^N$;
maximum order k of the functional mapping;
a tolerance ϵ to the duality gap.

Procedure :
compute the matrices of eigenvectors V_j and the vectors of eigenvalues λ_j of S_j, for $j = 1, \dots, N$;
compute the matrices of eigenvectors U_j and the vectors of eigenvalues φ_j of F_j, for $j = 1, \dots, N$;
set $c^\star = (L_v^\intercal L_v)^{-1} L_v^\intercal \varphi_v$ using L_v and φ_v defined in (19);
while $|\, p(Q^{(r)}) - q^\star| > \epsilon$
    compute $Q^{(r+1)} = \mathcal{L}(Q^{(r)})$, where $\mathcal{L}$ is the iterative procedure in [6];
end.

Output :
mapping of the functional connectivity matrix $\hat{F}_j = Q^\star \left(\sum_{r=0}^k c_r \operatorname{diag}(\lambda_j^r)\right) (Q^\star)^\intercal$.

4 Supplementary methods: AAL-parcellated dataset

In this section, we describe the methodological procedures conducted to obtain structural and functional brain scans for the dataset [18] following the AAL parcellation [30].

4.1 Study subjects

A total number of 133 people were designated to participate in this study. 38 of the subjects were not used in the final analysis due to the following reasons: 8 subjects did not pass MRI safety screening measures; 4 subjects were claustrophobic; 5 subjects had a technical error in data collection; 20 subjects missed more than 40 trials (over 10% of the trials) in either the memory for words or memory for faces test; 1 subject did not follow task instructions. Of the remaining 95 subjects, 11 had missing or unusable diffusion imaging data. We therefore analyzed data from 84 subjects: ages 27-45 (mean 34.21, standard deviation 4.23); 4 females, 80 males; 12 left-handed. None of the subjects were color blind. Informed written consent was obtained from each subject prior to the experimental sessions. All procedures were carried out in accordance with relevant guidelines and regulations, and were approved by the University of California, Santa Barbara Human Subjects Committee.

4.2 MRI data acquisition

Functional and structural scans were acquired using a research-dedicated, phased array 3T Siemens TIM Trio with a standard 12 channel head coil. Cushions were placed around the head to minimize head motion. Subjects held in their right hand an MRI compatible, two-button response box and in their left hand a squeeze ball for emergency purposes.

Functional Scans. Functional runs consisted of a T2*-weighted single shot gradient echo, echo-planar sequence sensitive to BOLD contrast (TR = 2.5s, TE = 30ms, FA = 90°) with generalized autocalibrating partially parallel acquisitions (GRAPPA; 64×64 matrix, 192mm×192mm FOV). Each volume consisted of 37 slices acquired parallel to the AC-PC plane, although the angle was slightly adjusted to optimize for frontal acquisition if necessary (interleaved, 3mm thickness with .5mm gap; 3mm×3mm in-plane resolution). Each functional scanning run began with the acquisition of four dummy volumes used to achieve steady-state tissue magnetization, which were later discarded. A transistor-transistor logic (TTL) pulse sent out by the

scanner at the onset of each viable EPI volume triggered the stimulus presentation software, and synchronized the beginning of each trial to the onset of the next complete image volume.

Once each subject was positioned in the scanner, anatomical and memory task functional scans were acquired first, followed by three blocks of attention task trials. A total of 146 volumes was acquired during the resting-state scan, for which subjects were instructed to remain still, keep their eyes open, and look at the blank screen. Note that these resting-state scans are similar in length to those used in previous studies to identify meaningful resting state networks [19, 4, 10, 22], and they are significantly longer than those used to examine the temporal evolution of brain network dynamics [19, 5]. A total of 540 volumes was acquired for each of the two memory test runs (faces and words), each consisting of 360 stimulus trials and 180 fixation trials. A total of 240 volumes was acquired for each of two attention task runs. The details of the experimental setup, stimuli, and testing conditions for the memory and attention tasks are described in the subsequent sections.

Structural Scans. Structural scans were acquired between memory test runs (see "Memory Tasks; Overall Procedure") using an echo planar diffusion weighted technique acquired with iPAT and an acceleration factor of 2. The timing parameters of the pulse sequence were TE/TR = 94/8400ms, 30 diffusion directions with a maximal b-value of 1000s/mm^2 and two averages. Two b0 images were acquired during the DTI scan. The matrix size was 128$\times$128 and the slice number was 60. The field of view was 230$\times$230mm^2 and the slice thickness 2mm. The acquisition time per DTI scan was 9:08min. In addition to the diffusion scan, a three dimensional (3D) high-resolution T1-weighted sagittal sequence image of the whole brain was obtained by a magnetization prepared rapid acquisition gradient-echo (MPRAGE) sequence with the following parameters: TR = 2300; TE = 2.98ms; flip angle = 9°; 160 slices; 1.10mm thickness.

4.3 Connectivity estimates

Functional connectivity was estimated from fMRI measurements acquired during resting state. Structural connectivity was estimated from the tractographic reconstruction of white matter pathways obtained from DTI measurements.

4.3.1 Functional connectivity

fMRI data preprocessing. Translational and rotational head movement was consistently small across subjects and datasets. The distributions of maximal translation and rotation from TR to TR were the same for rest, attention, memory for faces, and memory for word runs, with mean translational (μT) and rotational (μR) values of μT = [0.0787$\pm$0.0039mm, 0.0719$\pm$0.0043mm, 0.0513$\pm$0.0030mm, 0.0536$\pm$0.0037mm] and μR = [0.0493$\pm$0.0027°, 0.0503$\pm$ 0.0041°, 0.0362$\pm$0.0022°, 0.0391$\pm$0.0029°], reported here with the standard error of the mean. The largest maximum values of translation and rotation, measured across subjects and datasets, were 0.34mm and 0.33°, respectively. Functional images were realigned to correct for these head movements by registering all images to the first image in the time series. To further correct for noise-driven demagnetization and signal loss, heavily noisy task-related images with large global residuals were given decreased weight in the generalized linear model (as implemented via the robust weighted least squares toolbox, http://www.icn.ucl.ac.uk/motor control/imaging/robustWLS.html). Functional images were then coregistered to the anatomical image. The anatomical image was normalized, using combined segmentation and normalization, to conform to the Montreal Neurological Institute (MNI)152 template. The parameters of this transformation were applied to the functional images, which were re-sampled to 2mm isotropic voxels. Functional images were then smoothed using an isotropic Gaussian kernel (FWHM = 8mm). BOLD signals arising from any cerebrospinal fluid (CSF) or white matter tissue within the predominantly gray matter regions defined by the AAL atlas were not masked by each subject's anatomical MRI.

Functional connectivity. Average time series were extracted for each subject from 600 anatomical regions of interest (ROIs) defined by an upsampled version of the AAL atlas [31] covering the whole brain and including cortical and subcortical regions but excluding the cerebellar regions and vermis (see subsequent section on 'Atlases and Upsampling Procedure'). We focused our investigation on low frequency (0.06-0.125 Hz) oscillations in the BOLD signal that have previously been shown to support both resting [22, 4] and task-based connectivity [5]. The frequency band of interest was isolated by applying the maximal overlap discrete wavelet transform to each time series [27] and selecting scale 2 wavelet coefficients. We estimated the functional connectivity by computing the absolute value of the Pearson's correlation between all possible pairs of time series, creating a 600×600 ($M \times M$) connectivity matrix.

4.3.2 Structural connectivity

Diffusion data preprocessing. Data preprocessing and tractography were consistent with that reported in [2]. Motion artifact and image distortions caused by eddy-currents were corrected in FMRIB's Diffusion Toolbox in FSL software by applying an affine alignment of each diffusion-weighted image to the b0 image. In the current study, we did not correct for EPI distortions. In this Siemens scanner, the geometric distortion for diffusion imaging from EPI was found in prior tests to be less than 2mm (i.e., less than a single voxel) and was identified primarily along the anterior posterior (phase encoding) direction. Because the resolution of the diffusion images was larger than the magnitude of the distortion, no correction was required.

Tractography. Reconstruction of the diffusion images was performed using Diffusion Toolkit (DTK) (Ruopeng Wang, Van J. Wedeen, TrackVis.org, Martinos Center for Biomedical Imaging, Massachusetts General Hospital (MGH) [34]), a recently constructed software toolbox that provides precise diffusion imaging analysis and visualization capabilities [16, 35, 33, 32, 21, 26]. Diffusion tensor estimation was performed using the linear least-squares fitting method [34]. Raw data was not smoothed or sharpened prior to reconstruction. Deterministic tractography was subsequently performed in TrackVis software using the Fiber Assignment by Continuous Tracking (FACT) algorithm [24, 25, 36]. In this process, a single seed was placed in the center of each voxel, and the path was continued into the adjacent voxel that minimized the path curvature. Paths were terminated for curvatures greater than 35°. Fiber tracts that were rejected by the algorithm, such as those rejected due to high curvature, were not included in the present analysis. No further parameter tuning was performed within the software toolbox. Importantly, in using DTK, we performed an exhaustive search approach in which fiber tracking was performed within all voxels rather than within a set of regions specified a priori. Inter-regional connectivity was then examined by applying a set of gray matter masks to the complete tractography solution and by quantifying the number and length of tracts that passed between any two masks.

Inter-regional connectivity. In order to attain regional, rather than voxel-based, connectivity estimates, a set of M brain region masks were applied to the reconstructed fiber tracts using the UCLA Multimodal Connectivity Package (UMCP). We extracted two separate regional connectivity matrices: the total number and average length, as defined below. For all possible pairs of M masks, we determined the number of tracks originating in mask i and terminating in mask j ($i \neq j$), thereby creating an $M \times M$ inter-regional anatomical connectivity matrix, N, where each element N_{ij} specifies the number of tracts originating in mask i and terminating in mask j. The matrix sum of the connectivity matrix is the total number of reconstructed fiber tracts, F , between grey matter masks: $F = \sum_{i \neq j} N_{ij}$. The number of fiber tracts F uncovered by the algorithm was data driven rather that defined a priori and was therefore variable from subject to subject. In addition to the connectivity matrix, we determined the tract length matrix, L, where each element L_{ij} specifies the average length of the tracts originating in mask i and terminating in mask j.

4.4 Cortical parcellation

4.4.1 Brain region masks

Masks for each of the M regions of a cortical atlas were transformed into the subject's native space in a multi-step process [13, 28]. The subject's MPRAGE scans from the three sessions were averaged together to create a mean structural scan, which was then registered to the subject's b0 diffusion image using the affine transform provided by FSL's linear registration toolbox, FLIRT. This native space MPRAGE was then registered to the nonlinear MNI 152 T1 2mm brain. The inverse of this transformation matrix was then used to warp the atlas ROIs into subject-specific native space. The use of the MPRAGE as an intermediary between the native space and MNI space significantly increased registration fidelity and reproducibility of subsequently measured network properties.

4.4.2 Atlases and upsampling procedure

We employed an upsampled version of the original Automated Anatomical Labeling Atlas (AAL) containing 90 cortical and subcortical regions of interest but not containing cerebellar structures or the brainstem [31, 8]. This resampled version contained 600 regions of interest and was created via a series of upsampling steps in which any given region was bisected perpendicular to its principal spatial axis in order to create 2 equally sized sub-regions. Larger and smaller AAL regions were respectively upsampled more less and less in an effort to create an atlas of roughly equally-sized regions that still obeyed gross anatomical boundaries. The final atlas contained regions of an average size of 268 voxels (with a standard deviation of 35 voxels). Our choice of atlases was motivated by current work in the field. Several recent studies have highlighted the effect of region size on structural connectivity estimates [17, 2]. These results suggest that findings drawn from whole-brain atlases with highly variable region size must be interpreted carefully. One alternative approach is to employ a parcellation scheme of identically-sizes regions placed in a grid-like fashion across the brain (e.g. [11]). However, this approach selects regions of interest that can lie across important anatomical boundaries such as gyri and sulci. To address this issue, we have chosen to create an atlas of 600 regions with roughly uniform size, none of which cross the anatomical boundaries specified by the original AAL atlas. Regions within our 'uniform-600' atlas are therefore approximately equal in size while remaining anatomically constrained.

4.5 Connectivity matrices

Structural brain graphs were estimated via deterministic tractography applied on diffusion tensor imaging (DTI) scans [14]. Each region in the structural graph corresponds to a localized brain area in the Automated Anatomical Labeling (AAL) atlas [30]. In our experiments, we consider structural graphs with weighted edges using two types of connectivity: *Connect* and *Fractional Anisotropy* (FA). In the Connect type, the weight of an edge is given by the total number of white matter fiber tracts connecting a pair of brain regions. In the FA type, edge weights are computed using the fractional anisotropy of all voxels in the DTI scan, which is a scalar value describing the degree of anisotropy of the diffusion of water molecules in the voxel. The weight of an edge connecting two regions is, thereby, defined as the average value of the fractional anisotropy over the white matter streamlines connecting them [14]. Besides the structural graphs, we also consider functional connectivity matrices, which are built as follows. For each brain region in the AAL atlas, we extract a representative time-series using the scale-2 wavelet coefficients (0.06–0.125 Hz) of the mean BOLD signal in each region [3]. The (i, j)-th entry of the functional connectivity matrix is given by the Pearson's correlation coefficients between the representative time-series of regions i and j. Notice that the diagonal entries of the functional connectivity matrices are always equal to one. In our evaluations, we use structural and functional connectivity matrices obtained

from 84 different subjects measured non-invasively while at rest.

5 Supplementary results: AAL-parcellated dataset

In this section, we report the results of the individual spectral mapping when AAL-parcellated dataset is considered. This dataset employs legacy single-tensor diffusion technology, which imposes significant limitations[29, 20] with respect to the tractography and estimation of structural connectivity matrices. These findings are therefore included exclusively to enable discretionary comparison with previous studies that use comparably limited datasets. Specifically, in Section 5.1, we present mapping results obtained using the individual and group functional mapping, and, in Section 5.2, we show that the individual functional mapping is robust with respect to perturbations in the structural connectivity matrix.

5.1 Recovered connectivity matrices

We represent the evolution of the correlation quality between the mapped and the actual functional connectivity matrices when we vary the maximum length of the walks under consideration (denoted by the parameter k) for the individual and the group spectral mappings, which results are reported in Fig. S6. These cases are evaluated for the AAL-90 parcellation in (A-B), and for the AAL-600 parcellation in (C-D). Additionally, for the sake of illustration, in this subsection we present the connectivity matrices recovered by the individual spectral mapping for the AAL-90 and AAL-600 parcellations. More precisely, we consider the individual whose mapped functional connectivity matrix achieve a median correlation quality. In particular, in Fig. S7 we consider the AAL-90 parcellation, and we depict in (A) the structural connectivity matrix for the FA type, in (B) we display the in-sample functional connectivity matrix, in (C) we present the corresponding the out-of-sample matrix, and in (D) we show the mapped functional connectivity matrix using the individual spectral mapping with the structural connectivity matrix presented in (A). In Fig. S8, we present analogous results for the case where the FA structural type and the AAL-600 parcellation is considered.

5.2 Perturbation analysis

Now, we propose to investigate the robustness of the individual spectral mapping with respect to noise in the structural matrices. First, for each structural and functional connectivity matrix pair (S, F) associated with a given individual, we obtain the set of parameters $(a^\star, R^\star)$ obtained from Algorithm 1. Then, we generate a perturbed matrix $\check{S}$ from the original structural matrix S, i.e., we adopt a random multiplicative model acting on each element of the structural matrix. This model has the desirable property of preserving the structural matrix sparsity, and producing perturbations which are, on average, proportional to the value of each element. Quantitatively, the perturbation model can be described as follows. For each element $[S]_{ij}$ or the original structural matrix, we sample a perturbation value from a uniform distribution with support between $(-\rho, \rho)$, i.e., $[\Delta]_{ij} \sim$ uniform$(-\rho, \rho)$, where ρ is a parameter that we vary. An entry of the perturbed matrix is, therefore, defined as

$$[\check{S}]_{ij} = (1 + [\Delta]_{ij})[S]_{ij}. \tag{20}$$

Subsequently, we compute the (perturbed) mapped functional connectivity matrix $\check{F}$ using the set of parameters $(a^\star, R^\star)$, i.e.,

$$\check{F} = R^* \left(\sum_{i=0}^{k} a_i^* \check{S}^i \right) (R^*)^\intercal.$$

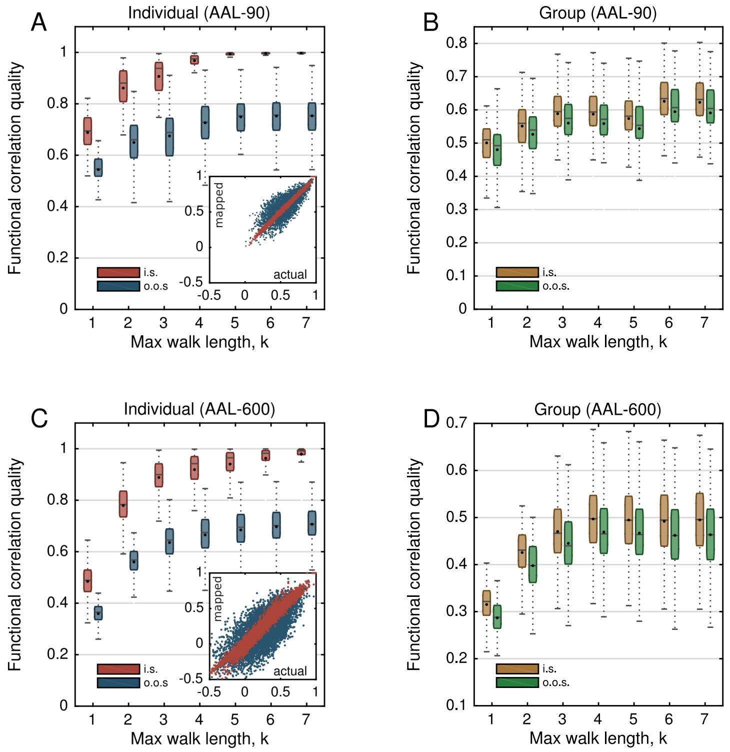

Fig. S6 Spectral mapping performance (FA type). We represent the evolution of the correlation quality between the mapped and the actual functional connectivity matrices when we vary the maximum length of the walks under consideration (denoted by the parameter k) for the individual and the group spectral mappings. These cases are evaluated for the AAL-90 parcellation in (A-B), and for the AAL-600 parcellation in (C-D), considering both the in-sample (i.s.) and out-of-sample (o.o.s.) scenarios. In (A) and (C), we plot the evolution of the correlation quality evaluated over 10 different splits of the BOLD signal time-series wavelet coefficients in the training (in red) and validation (in blue) sets. The inset plots in (A) and (C) include scatter plots of the entries of the mapped functional matrix $\hat{F}$ (values in the ordinates) versus the actual connectivity matrix F (values in the abscissae) when $k = 5$ for the individual with the median correlation quality. The ordinates of the red (respectively, blue) dots in the scatter plot correspond to the entries of the functional connectivity matrix used for training (respectively, validation). In (B) and (D), we plot the evolution of the correlation quality evaluated over 10 different splits of the BOLD signal time-series in the training (in yellow) and validation (in green) sets.

Finally, in order to assess the robustness of the individual spectral mapping, we compare the functional correlation quality ucorr $(\hat{F}, \check{F})$ for all individuals and different values of parameter ρ, the results of which we present in Fig. S9, for the Connect structural type. In particular, we consider the parameter $\rho = \{0.20, 0.40\}$ and generate 100 perturbation matrices for each individual with entries obtained according to (20). Take the parameter $k = 5$. In the FA structural type, the out-of-sample functional correlation achieved was 0.749 for the unperturbed case, whereas we achieve 0.769 and 0.745 for the perturbed case with $\rho = 0.20$ and $\rho = 0.40$, respectively. In conclusion, these results support the fact that the proposed mapping is robust with respect perturbations in the structural matrix.

6 Comparison with baseline methods

In this section, we briefly describe and compare two alternative methods to solve the individual mapping problem: (i) the method proposed in [15], which we refer to as the *DKHM method* and present in Section 6.1; and (ii) the method proposed in [23], which

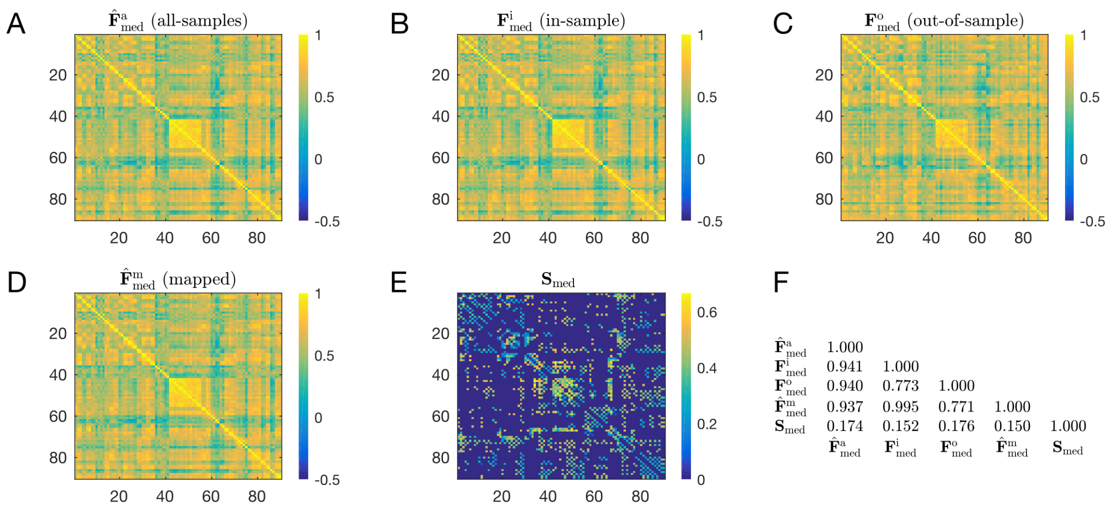

Fig. S7 Structural (FA type) and functional connectivity matrices for the AAL-90 parcellation. We consider an individual whose mapped functional connectivity achieves the median correlation quality across all subjects, for parameter $k = 5$. In (A), we present the functional connectivity matrix obtained from all time samples. In (B) and (C), we show the empirical functional connectivity matrix for the same individual obtained from the in-sample and out-of-sample data, respectively. In (D) we display the mapped functional connectivity matrix using the individual spectral mapping, whereas in (E) we present its structural connectivity matrix. Lastly, (F) displays the pair-wise correlation between the above mentioned matrices, for all possible combinations.

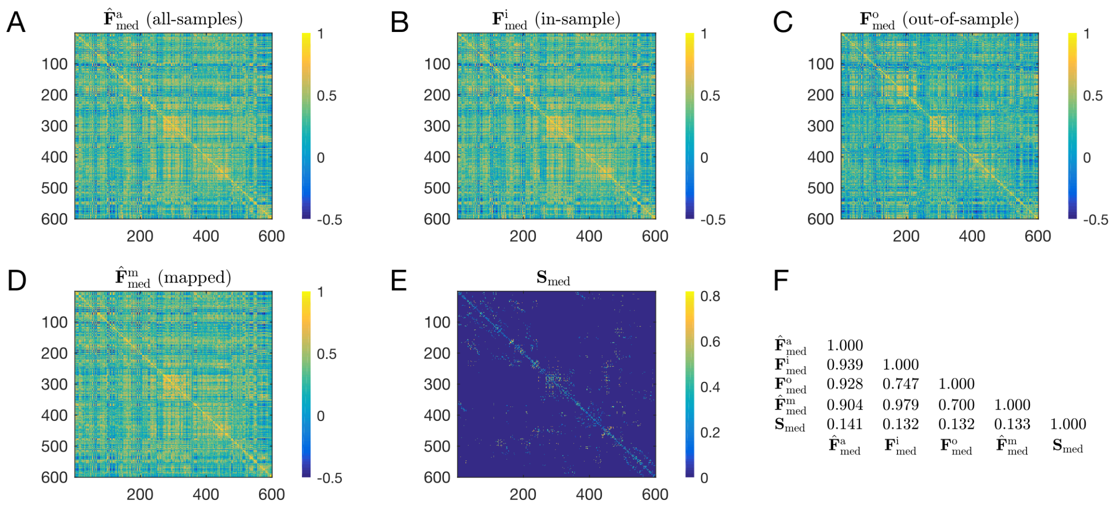

Fig. S8 Structural (FA type) and functional connectivity matrices for the AAL-600 parcellation. We consider an individual whose mapped functional connectivity achieves the median correlation quality across all subjects, for parameter $k = 5$. In (A), we present the functional connectivity matrix obtained from all time samples. In (B) and (C), we show the empirical functional connectivity matrix for the same individual obtained from the in-sample and out-of-sample data, respectively. In (D) we display the mapped functional connectivity matrix using the individual spectral mapping, whereas in (E) we present its structural connectivity matrix. Lastly, (F) displays the pair-wise correlation between the above mentioned matrices, for all possible combinations.

we refer to as *Taylor Approximation* and present in Section 6.2

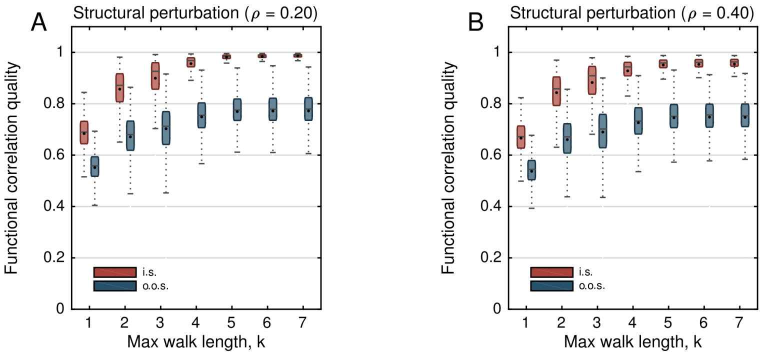

Fig. S9 Robustness of the mapped functional connectivity to perturbations in the structural connectivity matrix (FA type, AAL-90). In (A) and (B), we present the distribution of functional correlation quality of the mapped functional connectivity matrix as a function of the maximum walk length when the individual's structural connectivity matrix has its entries perturbed by at most 20% ($\rho = 0.2$) and 40% ($\rho = 0.4$), respectively.

6.1 DKHM

The DKHM method was introduced to solve the mapping problem between the structural and functional matrices of a representative (or average) individual of a population (as illustrated in Fig. S3). In order to compare our mapping with that in [15], we apply the DKHM method to each individual in our dataset (as illustrated in Fig. S2).

The DKHM method builds a mapping of the average functional matrix based on a linear combination of four primitives: (*i*) transformed path weight, (*ii*) number of steps, (*iii*) search information, and (*iv*) path transitivity. In order to define these primitives, we need to introduce the following notions. Given an $n \times n$ matrix S, we define a graph with n nodes and a set of edges $E = \{\{i,j\} \colon [S]_{ij} > 0\}$, i.e, the pairs $\{i,j\}$ for which $[S]_{ij}$ is strictly positive. To each edge $\{i,j\} \in E$, we associate a weight $w_{ij} = [S]_{ij}$ and an inverse weight $w'_{ij} = w_{ij}^{-1}$. A path of length l from node i_0 to i_l is an ordered sequence of $l+1$ nodes, $(i_0, i_1, \ldots, i_l)$, such that $\{i_{r-1}, i_r\} \in E$ for all $r = 1, \ldots, l$. Given a path in the graph, we define its weight (respectively, inverse weight) as the sum of the weights (respectively, inverse weights) of the edges visited by the path. Given a pair of nodes s and t, the shortest path from s to t with respect to the inverse weight is denoted by $\mathcal{P}^*_{s\to t}$. We also denote the set of edges visited by $\mathcal{P}^*_{s\to t}$ by $\mathcal{E}^*_{s\to t}$. For each node i in the graph, we define its weighted out-degree as $w_i = \sum_{i\neq j} w_{ij}$. Then, for a pair of nodes s and t, we define the following quantity

$$P(\mathcal{P}^*_{s\to t}) = \prod_{\{i,j\}\in\mathcal{E}^*_{s\to t}} \frac{w_{ij}}{w_i}.$$

Furthermore, the *search information* h_{st} is defined as

$$h_{st} = \frac{1}{2}\left[-\log_2\left(P(\mathcal{P}^*_{s\to t})\right) - \log_2\left(P(\mathcal{P}^*_{t\to s})\right)\right], \tag{21}$$

which is a symmetric function, since $h_{st} = h_{ts}$.

We also need to introduce the *matching index* between a pair of nodes i and j, which is given by

$$\mu_{ij} = \frac{\sum_{k\neq i,j}(w_{ik} + w_{jk})\Theta(w_{ik})\Theta(w_{jk})}{\sum_{k\neq j} w_{ik} + \sum_{k\neq i} w_{jk}}, \tag{22}$$

where $\Theta(w_{ij}) = 1$ if $w_{ij} > 0$, and 0 otherwise. Averaging the matching index over all possible pairs of nodes along the shortest

path between s and t results in the so-called *path transitivity*, which is formally defined as

$$m_{st} = \frac{2}{|\mathcal{P}^*_{s\to t}|(|\mathcal{P}^*_{s\to t}|-1)} \sum_{i\in\mathcal{P}^*_{s\to t}} \sum_{j\in\mathcal{P}^*_{s\to t}} \mu_{ij}, \tag{23}$$

where edges in the shortest path are excluded.

Finally, since each primitive is a function of a pair of nodes, they can be represented via matrices, as follows. The $n \times n$ matrix K contains shortest path lengths between all pairs of nodes, i.e., $[K]_{ij}$ is the length of the shortest path between nodes i and j. Also, the $n \times n$ matrix D contains the inverse weights of the shortest paths. In addition, the search information between all pairs of nodes is captured by the $n \times n$ matrix H, where $[H]_{ij} = h_{ij}$. Finally, the $n \times n$ matrix M contains path transitivities between all pairs of nodes, i.e., $[M]_{ij} = m_{ij}$.

In [15], the authors proposed to linearly combine the four primitives described above to form a mapping of the functional connectivity matrix. We can mathematically describe this mapping as follows. Consider the $\binom{n}{2} \times 5$ matrix $G = [\,\mathrm{vec}(D)\,|\,\mathrm{vec}(K)\,|\,\mathrm{vec}(H)\,|\,\mathrm{vec}(M)\,|\,\mathrm{vec}(1_n 1_n^\intercal)\,]$, i.e., the first four columns of G are vectorized versions of the primitives and the last column contains all ones. These primitives are linearly combined using the vector of weights $w^\star \in \mathbb{R}^5$ which solves the following optimization problem:

$$\underset{w}{\text{minimize}} \quad \|\mathrm{vec}(F) - G\,w\|_2^2\,. \tag{24}$$

The solution $w^\star$ to (24) is obtained as follows:

$$w^\star = (G^\intercal G)^{-1} G^\intercal \mathrm{vec}(F). \tag{25}$$

Consequently, the functional connectivity estimate $\hat{F}$ for a specific individual satisfies $\mathrm{vec}(\hat{F}) = G w^\star$, from which the $n \times n$ matrix $\hat{F}$ can be retrieved by reverting the vectorization operation. Finally, the results for the individual mapping are depicted in Fig. S10, which clearly shows that our method outperforms the results attained by the DKHM method.

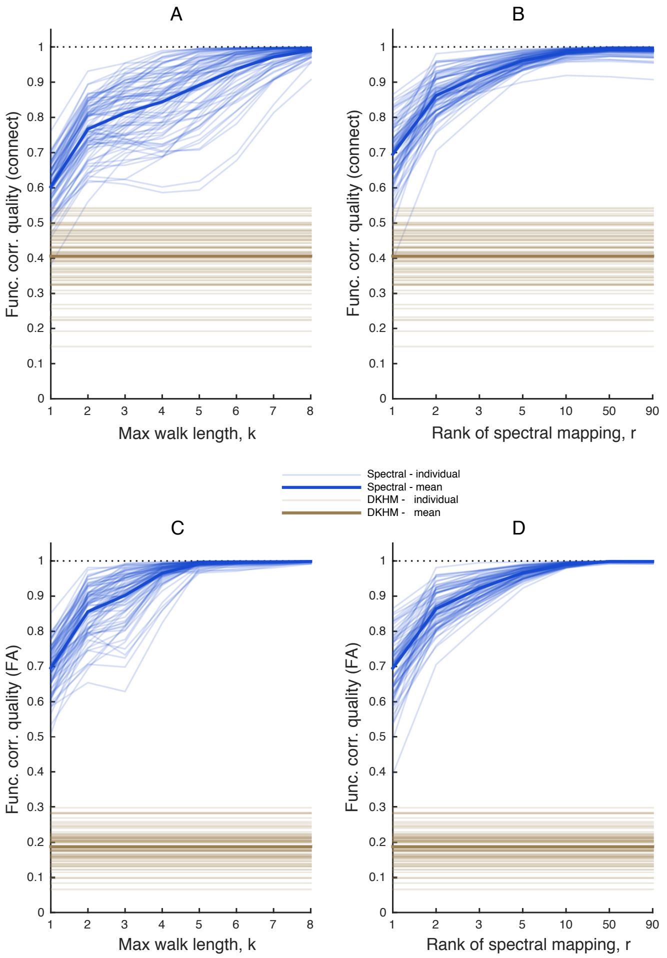

Fig. S10 Comparison of the individual spectral mapping with the DKHM method. Each light-blue line in the above plots represents the functional correlation quality for each one of the 84 individuals in the dataset as we vary k (A, C), or r (B, D). Brown lines correspond to the functional correlation quality of each subject obtained from the benchmark DKHM method (notice that these values are independent of k and r). In (A, B), we use the Connect connectivity in our comparison, while in (C, D) we use the FA connectivity. Panels on the left (A, C) display results for different values of k when $r = n$. Panels on the right (B, D) present the results for different values of r when $k = 7$.

6.2 Taylor Approximation

In [23], the authors assume that the functional connectivity is described as a nonlinear mapping of the structural connectivity. Let $F_j \in \mathbb{R}^{n\times n}$ be the functional connectivity matrix for individual j, and $S_j \in \mathbb{R}^{n\times n}$ its structural connectivity matrix. Also, let the nonlinear mapping be described by $f_j : \mathbb{R}^{n\times n} \to \mathbb{R}^{n\times n}$, then we obtain

$$F_j = f_j(S_j). \tag{26}$$

Assuming that f_j is analytical, it can be locally approximated in a point $X_0 \in \mathbb{R}^{n\times n}$ as follows

$$f_j(S_j) = \sum_{i=0}^{\infty} c_i (S_j - X_0)^i, \tag{27}$$

where $c_i \in \mathbb{R}$ depends on the derivatives of f_j. Subsequently, the authors assume that (26) can be approximated by a finite truncation of (27) around the origin, i.e., $X_0 = 0$. Hence, in [23], the authors propose the following mapping:

$$\hat{F}_j = \sum_{i=0}^{k} c_i^\star (S_j)^i + c_{-1}^\star 1_n 1_n^\intercal, \tag{28}$$

where $\{c_i^\star\}_{i=-1}^{k}$ are the components of the vector $c = (c_{-1}^\star, c_0^\star, \ldots, c_k^\star) \in \mathbb{R}^{k+2}$ that solve the following optimization problem

$$\underset{c}{\text{minimize}} \quad \|\text{vec}(F_j) - G_j c\|_2^2 \,, \tag{29}$$

with $G_j = \left[\, \text{vec}(1_n 1_n^\intercal) \,|\, \text{vec}(I_n) \,|\, \text{vec}(S_j) \,|\, \ldots \,|\, \text{vec}(S_j^k) \,\right]$. Therefore the optimal solution can be obtained as

$$c^\star = (G_j^\intercal G_j)^{-1} G_j^\intercal \text{vec}(F_j).$$

Although (28) is similar to the individual spectral mapping proposed by us (see Section 3.1), the following remarks are due. First, we notice that the derivation proposed to obtain (28) is different from the one proposed by us, in that it considers an offset term $c_{-1}^\star 1_n 1_n^\intercal$ and does not consider a rotation matrix. Secondly, and most importantly, these differences are significant when computing the performance of the mappings, as we report in Fig. S11. Specifically, for a maximum walk length $k = 5$, the in-sample mean correlation coefficients attained using this method are 0.317 and 0.249 in average for the Connect and FA respectively. These contrast with the corresponding mean coefficient values of 0.898 and 0.993 when the Individual Spectral method is considered, see Fig. 4 and Fig. S6. Similarly, the out-of-sample mean correlation coefficients attained using the Taylor approximation method are 0.311 and 0.240 the Connect and FA respectively, contrasting with 0.710 and 0.749 for the Individual Spectral method. In conclusion, the proposed individual spectral mapping mapping outperforms the mapping in (28) when either Connect or FA structural connectivity types are considered.

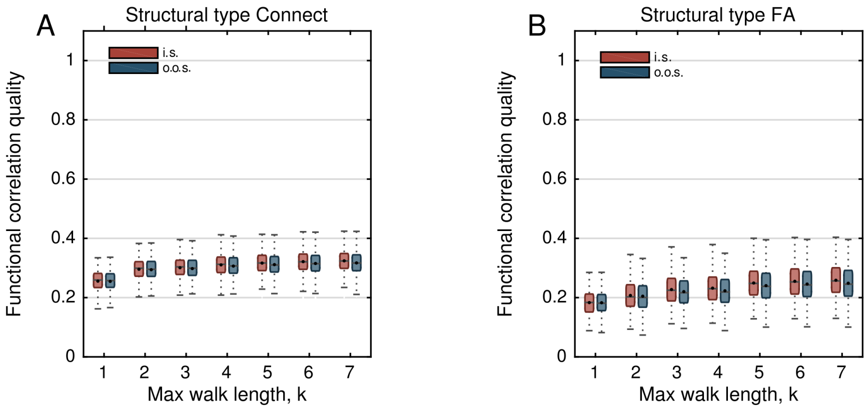

Fig. S11 Functional correlation quality using Taylor approximation (AAL-90). In (A) and (B), we present the distribution of functional correlation quality of the mapped functional connectivity matrix as a function of the maximum walk length for the Connect and FA structural types, respectively.

References

[1] P.-A. Absil, R. Mahony, and R. Sepulchre. *Optimization algorithms on matrix manifolds*. Princeton University Press, 2009.

[2] D. S. Bassett, J. A. Brown, V. Deshpande, J. M. Carlson, and S. T. Grafton. Conserved and variable architecture of human white matter connectivity. *Neuroimage*, 54(2):1262–1279, 2011.

[3] D. S. Bassett, J. A. Brown, V. Deshpande, J. M. Carlson, and S. T. Grafton. Conserved and variable architecture of human white matter connectivity. *Neuroimage*, 54(2):1262–1279, 2011.

[4] D. S. Bassett, B. G. Nelson, B. A. Mueller, J. Camchong, and K. O. Lim. Altered resting state complexity in schizophrenia. *Neuroimage*, 59(3):2196–2207, 2012.

[5] D. S. Bassett, N. F. Wymbs, M. A. Porter, P. J. Mucha, J. M. Carlson, and S. T. Grafton. Dynamic reconfiguration of human brain networks during learning. *Proceedings of the National Academy of Sciences*, 108(18):7641–7646, 2011.

[6] N. Boumal, B. Mishra, P.-A. Absil, and R. Sepulchre. Manopt, a Matlab toolbox for optimization on manifolds. *The Journal of Machine Learning Research*, 15(1):1455–1459, 2014.

[7] S. Boyd and L. Vandenberghe. *Convex optimization*. Cambridge University Press, 2004.

[8] R. S. Desikan, F. Ségonne, B. Fischl, B. T. Quinn, B. C. Dickerson, D. Blacker, R. L. Buckner, A. M. Dale, R. P. Maguire, B. T. Hyman, et al. An automated labeling system for subdividing the human cerebral cortex on mri scans into gyral based regions of interest. *Neuroimage*, 31(3):968–980, 2006.

[9] A. Edelman, T. A. Arias, and S. T. Smith. The geometry of algorithms with orthogonality constraints. *SIAM journal on Matrix Analysis and Applications*, 20(2):303–353, 1998.

[10] Q. Fang, S. Sakadžić, L. Ruvinskaya, A. Devor, A. M. Dale, and D. A. Boas. Oxygen advection and diffusion in a three-dimensional vascular anatomical network. *Optics express*, 16(22):17530–17541, 2008.

[11] A. Fornito, A. Zalesky, D. S. Bassett, D. Meunier, I. Ellison-Wright, M. Yücel, S. J. Wood, K. Shaw, J. O'Connor, D. Nertney, et al. Genetic influences on cost-efficient organization of human cortical functional networks. *The Journal of Neuroscience*, 31(9):3261–3270, 2011.

[12] W. Fulton and J. Harris. *Representation theory*, volume 129. Springer Science & Business Media, 1991.

[13] G. Gong, Y. He, L. Concha, C. Lebel, D. W. Gross, A. C. Evans, and C. Beaulieu. Mapping anatomical connectivity patterns of human cerebral cortex using in vivo diffusion tensor imaging tractography. *Cerebral cortex*, 19(3):524–536, 2009.

[14] G. Gong, Y. He, L. Concha, C. Lebel, D. W. Gross, A. C. Evans, and C. Beaulieu. Mapping anatomical connectivity patterns of human cerebral cortex using in vivo diffusion tensor imaging tractography. *Cerebral Cortex*, 19(3):524–536, 2009.

[15] J. Goñi, M. P. van den Heuvel, A. Avena-Koenigsberger, N. V. de Mendizabal, R. F. Betzel, A. Griffa, P. Hagmann, B. Corominas-Murtra, J.-P. Thiran, and O. Sporns. Resting-brain functional connectivity predicted by analytic measures of network communication. *Proceedings of the National Academy of Sciences*, 111(2):833–838, 2014.

[16] C. Granziera, J. D. Schmahmann, N. Hadjikhani, H. Meyer, R. Meuli, V. Wedeen, and G. Krueger. Diffusion spectrum imaging shows the structural basis of functional cerebellar circuits in the human cerebellum in vivo. *PLoS One*, 4(4):e5101, 2009.

[17] P. Hagmann, L. Cammoun, X. Gigandet, R. Meuli, C. J. Honey, V. J. Wedeen, and O. Sporns. Mapping the structural core of human cerebral cortex. *PLoS Biol*, 6(7):e159, 2008.

[18] A. M. Hermundstad, D. S. Bassett, K. S. Brown, E. M. Aminoff, D. Clewett, S. Freeman, A. Frithsen, A. Johnson, C. M. Tipper, M. B. Miller, et al. Structural foundations of resting-state and task-based functional connectivity in the human brain. *Proceedings of the National Academy of Sciences*, 110(15):6169–6174, 2013.

[19] D. T. Jones, P. Vemuri, M. C. Murphy, J. L. Gunter, M. L. Senjem, M. M. Machulda, S. A. Przybelski, B. E. Gregg, K. Kantarci, D. S. Knopman, et al. Non-stationarity in the âĂIJresting brainâĂŹsâĂİ modular architecture. *PloS one*, 7(6):e39731, 2012.

[20] T. R. Knösche, A. Anwander, M. Liptrot, and T. B. Dyrby. Validation of tractography: Comparison with manganese tracing. *Human brain mapping*, 36(10):4116–4134, 2015.

[21] M. Lagana, M. Rovaris, A. Ceccarelli, C. Venturelli, S. Marini, and G. Baselli. Dti parameter optimisation for acquisition at 1.5 t: Snr analysis and clinical application. *Computational intelligence and neuroscience*, 2010:8, 2010.

[22] M.-E. Lynall, D. S. Bassett, R. Kerwin, P. J. McKenna, M. Kitzbichler, U. Muller, and E. Bullmore. Functional connectivity and brain networks in schizophrenia. *The Journal of Neuroscience*, 30(28):9477–9487, 2010.

[23] J. Meier, P. Tewarie, A. Hillebrand, L. Douw, B. W. van Dijk, S. M. Stufflebeam, and P. Van Mieghem. A mapping between structural and functional brain networks. *Brain connectivity*, 6(4):298–311, 2016.

[24] S. Mori, B. J. Crain, V. Chacko, and P. Van Zijl. Three-dimensional tracking of axonal projections in the brain by magnetic resonance imaging. *Annals of neurology*, 45(2):265–269, 1999.

[25] S. Mori and P. van Zijl. Fiber tracking: principles and strategies–a technical review. *NMR in Biomedicine*, 15(7-8):468–480, 2002.

[26] M. Nezamzadeh, V. J. Wedeen, R. Wang, Y. Zhang, W. Zhan, K. Young, D. J. Meyerhoff, M. W. Weiner, and N. Schuff. In-vivo investigation of the human cingulum bundle using the optimization of mr diffusion spectrum imaging. *European journal of radiology*, 75(1):e29–e36, 2010.

[27] D. B. Percival and A. T. Walden. *Wavelet methods for time series analysis*, volume 4. Cambridge university press, 2006.

[28] N. Shu, Y. Liu, J. Li, Y. Li, C. Yu, and T. Jiang. Altered anatomical network in early blindness revealed by diffusion tensor tractography. *PloS one*, 4(9):e7228, 2009.

[29] C. Thomas, Q. Y. Frank, M. O. Irfanoglu, P. Modi, K. S. Saleem, D. A. Leopold, and C. Pierpaoli. Anatomical accuracy of brain connections derived from diffusion mri tractography is inherently limited. *Proceedings of the National Academy of Sciences*, 111(46):16574–16579, 2014.

[30] N. Tzourio-Mazoyer, B. Landeau, D. Papathanassiou, F. Crivello, O. Etard, N. Delcroix, B. Mazoyer, and M. Joliot. Automated anatomical labeling of activations in SPM using a macroscopic anatomical parcellation of the MNI MRI single-subject brain. *Neuroimage*, 15(1):273–289, 2002.

[31] N. Tzourio-Mazoyer, B. Landeau, D. Papathanassiou, F. Crivello, O. Etard, N. Delcroix, B. Mazoyer, and M. Joliot. Automated anatomical labeling of activations in spm using a macroscopic anatomical parcellation of the mni mri single-subject brain. *Neuroimage*, 15(1):273–289, 2002.

[32] M. Vishwas, T. Chitnis, R. Pienaar, B. Healy, and P. Grant. Tract-based analysis of callosal, projection, and association pathways in pediatric patients with multiple sclerosis: a preliminary study. *American Journal of Neuroradiology*, 31(1):121–128, 2010.

[33] M. Wahl, Z. Strominger, R. Jeremy, A. Barkovich, M. Wakahiro, E. Sherr, and P. Mukherjee. Variability of homotopic and heterotopic callosal connectivity in partial agenesis of the corpus callosum: a 3t diffusion tensor imaging and q-ball

tractography study. *American Journal of Neuroradiology*, 30(2):282–289, 2009.

[34] R. Wang, T. Benner, A. Sorensen, and V. Wedeen. Diffusion toolkit: a software package for diffusion imaging data processing and tractography. In *Proc Intl Soc Mag Reson Med*, volume 15, 2007.

[35] V. J. Wedeen, R. Wang, J. D. Schmahmann, T. Benner, W. Tseng, G. Dai, D. Pandya, P. Hagmann, H. D'Arceuil, and A. J. de Crespigny. Diffusion spectrum magnetic resonance imaging (dsi) tractography of crossing fibers. *Neuroimage*, 41(4):1267–1277, 2008.

[36] R. Xue, P. van Zijl, B. J. Crain, M. Solaiyappan, and S. Mori. In vivo three-dimensional reconstruction of rat brain axonal projections by diffusion tensor imaging. *Magnetic Resonance in Medicine*, 42(6):1123–1127, 1999.
